# Age and Genetic Risk Score and Rates of Blood Lipid Changes in China

**DOI:** 10.1001/jamanetworkopen.2023.5565

**Published:** 2023-03-29

**Authors:** Jianxin Li, Mengyao Liu, Fangchao Liu, Shufeng Chen, Keyong Huang, Jie Cao, Chong Shen, Xiaoqing Liu, Ling Yu, Yingxin Zhao, Huan Zhang, Shujun Gu, Liancheng Zhao, Ying Li, Dongsheng Hu, Jianfeng Huang, Dongfeng Gu, Xiangfeng Lu

**Affiliations:** 1 Department of Epidemiology, Fuwai Hospital, National Center for Cardiovascular Diseases, Chinese Academy of Medical Sciences and Peking Union Medical College, Beijing, China; 2 Key Laboratory of Cardiovascular Epidemiology, Chinese Academy of Medical Sciences, Beijing, China; 3 Department of Epidemiology, School of Public Health, Nanjing Medical University, Nanjing, China; 4 Division of Epidemiology, Guangdong Provincial People’s Hospital and Cardiovascular Institute, Guangzhou, China; 5 Department of Cardiology, Fujian Provincial Hospital, Fuzhou, China; 6 Cardio-Cerebrovascular Control and Research Center, Institute of Basic Medicine, Shandong Academy of Medical Sciences, Jinan, China; 7 Department of Epidemiology, School of public health, Medical College of Soochow University, Suzhou, China; 8 Department of Chronic Disease Control and Prevention, Changshu Center for Disease Control and Prevention, Changshu, China; 9 Department of Biostatistics and Epidemiology, School of Public Health, Shenzhen University Health Science Center, Shenzhen, China; 10 Department of Epidemiology and Health Statistics, College of Public Health, Zhengzhou University, Zhengzhou, China; 11 School of Medicine, Southern University of Science and Technology, Shenzhen, China; 12 National Clinical Research Center for Cardiovascular Diseases, State Key Laboratory of Cardiovascular Disease, Fuwai Hospital, National Center for Cardiovascular Diseases, Chinese Academy of Medical Sciences and Peking Union Medical College, Beijing, China

## Abstract

**Importance:**

Blood lipids are the primary cause of atherosclerosis. However, little is known about relationships between rates of blood lipid changes and age and genetic risk.

**Objective:**

To evaluate associations of blood lipid change rates with age and polygenic risk.

**Design, Setting, and Participants:**

This cohort is from the Prediction for Atherosclerotic Cardiovascular Disease Risk in China, which was established from 1998 to 2008. Participants were followed up until 2020 (mean [SD] follow-up, 13.8 [4.3] years) and received 4 repeated lipid measurements. Data analysis was performed from June to August 2022. A total of 47 691 participants with available genotype data were recruited, and 37 317 participants aged 18 years or older were included in the final analysis after excluding participants who were lost to follow-up or with major chronic diseases, and those without blood lipid measurements at baseline and any follow-up survey.

**Exposures:**

Age and polygenic risk scores based on 126 lipid-related genetic variants.

**Main Outcomes and Measures:**

The estimated annual changes (EAC) of blood lipids in milligrams per deciliter.

**Results:**

This study evaluated 37 317 participants (mean [SD] age of 51.37 [10.82] years; 15 664 [41.98%] were male). The associations of EACs of blood lipids with age differed substantially between male and female participants. Male participants experienced declining change as they got older for total cholesterol (EAC, 0.34 [95% CI, 0.14 to 0.54] mg/dL for age <40 years vs 0.01 [95% CI, −0.11 to 0.13] mg/dL for age ≥60 years), triglyceride (EAC, 3.28 [95% CI, 2.50 to 4.07] mg/dL for age <40 years vs −1.70 [95% CI, −2.02 to −1.38] mg/dL for age ≥60 years), and low-density lipoprotein cholesterol (LDL-C) (EAC, 0.15 [95% CI, −0.02 to 0.32] mg/dL for age <40 years vs 0.01 [95% CI, −0.10 to 0.11] mg/dL for age ≥60 years). Female participants had inverse V-shaped associations and the greatest rate of change appeared in the age group of 40 to 49 years (EAC for total cholesterol, 1.33 [95% CI, 1.22 to 1.44] mg/dL; EAC for triglyceride, 2.28 [95% CI, 1.94 to 2.62] mg/dL; and EAC for LDL-C, 0.94 [95% CI, 0.84 to 1.03] mg/dL). Change in levels of blood lipids were also associated with polygenic risk. Participants at low polygenic risk tended to shift toward lower blood lipid levels, with EACs of −0.16 (95% CI, −0.25 to −0.07) mg/dL; −1.58 (95% CI, −1.78 to −1.37) mg/dL; and −0.13 (95% CI, −0.21 to −0.06) mg/dL for total cholesterol, triglyceride, and LDL-C, respectively. Participants with high polygenic risk had the greatest rates of change for total cholesterol, triglyceride, and LDL-C (EAC, 1.12 [95% CI, 1.03 to 1.21] mg/dL; EAC, 3.57 [95% CI, 3.24 to 3.91] mg/dL; and EAC, 0.73 [95% CI, 0.65 to 0.81] mg/dL, respectively). Similar patterns were also observed across sex and age groups.

**Conclusions and Relevance:**

In this cohort study, EACs of blood lipids were significantly associated with age and polygenic risk, suggesting that prevention strategies for lipids should focus on individuals with high genetic risk and in the critical age window.

## Key Points

**Question:**

Are age and genetic risk associated with rates of blood lipid changes among adults in China?

**Findings:**

In this cohort study of 37 317 participants, the estimated annual changes of blood lipids were associated with age and polygenic risk. Moreover, the associations of the estimated annual lipid changes with age differed significantly between male and female participants.

**Meaning:**

These findings suggest that strategies for precision management of lipid levels should focus on individuals at high genetic risk and in the critical age window.

## Teaser

This cohort study evaluates the association of age and genetic risk score with annual rates of blood lipid changes among adults in China.

## Introduction

Blood lipids are the primary cause of atherosclerosis, which contributes most to atherosclerotic cardiovascular disease, the leading cause of death globally.^1,2^ High low-density lipoprotein cholesterol (LDL-C), the dominant form of atherogenic cholesterol, was responsible for 4.32 million deaths worldwide in 2017.^3^ Triglyceride (TG) also plays an important role in cardiovascular risk through the atherogenic indicator as TG-rich lipoprotein.^1,4,5^ Hence guidelines have highlighted that blood lipid control is the foundation of primary prevention of cardiovascular disease.^1,5,6,7^ Although declining trends in blood lipid levels have occurred in Western countries over the past 2 decades,^8^ the deteriorating trends persist in low- and middle-income countries due to unfavorable changes of lifestyle and environmental factors, especially in China.^9,10^ Therefore, it is critical to implement effective strategies for blood lipid management among the Chinese population.

Strategies for blood lipid control are typically focused on individuals who currently have high levels.^1,7^ However, it is hard to reverse the subsequent deleterious cardiovascular effects once the dyslipidemia emerges. Thus, earlier prevention is essential.^11^ The investigation of the dynamic trends in the blood lipid change profile over age in a population may help us determine the potentially critical period for prevention even when lipid levels are normal. Previous studies have evaluated the relationships between age and lipid levels,^12,13,14^ but the associations between blood lipid change rates and age are unknown. Therefore, it is urgent to examine the relationships of lipid change rates with age. It has been considered that blood lipid levels could be largely determined by genetic factors under the pattern of polygenic inheritance.^5^ We assumed that the lipid change profile could be influenced by genetic factors. Many genetic loci related to blood lipid levels have been identified by genome-wide association studies.^15,16,17,18,19^ It provides an opportunity to construct a polygenic risk score (PRS), a quantitative measure of inherited susceptibility by integrating all available lipid-associated genetic loci. The PRS is highly associated with blood lipid levels^20,21,22^ and is recommended as a potentially useful tool for risk assessment.^23,24^

Here, we first generated PRSs for 4 lipid levels by incorporating the lipid-related genetic loci from large-scale genome-wide association studies. Then we determined the rate pattern of blood lipid changes and further estimated the associations of the change rates with PRS in a large Chinese population-based longitudinal cohort.

## Methods

### Study Participants

Study participants came from 3 subcohorts of the Prediction for Atherosclerotic Cardiovascular Disease Risk in China (China-PAR) study, a large Chinese population-based longitudinal cohort study. The 3 subcohorts included China Multi-Center Collaborative Study of Cardiovascular Epidemiology (China MUCA) in 1998, International Collaborative Study of Cardiovascular Disease in Asia (InterASIA) from 2000 to 2001, and Community Intervention of Metabolic Syndrome in China and Chinese Family Health Study (CIMIC) from 2007 to 2008. The China MUCA and InterASIA studies were followed up from 2007 to 2008. Subsequently, all 3 subcohorts were followed up from 2012 to 2015 and 2018 to 2020. Details of the cohort have been described in eMethods in Supplement 1 and elsewhere.^25^ The China-PAR project was approved by the institutional review board at Fuwai Hospital in Beijing, China, and written informed consent was obtained from all participants. This study followed the Strengthening the Reporting of Observational Studies in Epidemiology (STROBE) reporting guideline for cohort studies.

In summary, among 47 691 participants aged 18 years or older with available genotype data in 3 subcohorts, 46 508 participants completed the follow-up examinations. We further excluded 1803 participants with major chronic diseases (myocardial infarction, stroke, heart failure, kidney failure, and cancer), 726 participants without blood lipid measurements at baseline examination, and 6662 participants without available lipid data at any follow-up survey. Finally, a total of 37 317 participants (mean [SD] follow-up of 13.8 [4.3] years) remained in the current analysis (eFigure 1 in Supplement 1). The included and excluded participants had no substantial difference in baseline characteristics, including blood lipid levels (eTable 1 in Supplement 1).

### Data Collection

Four examinations were conducted per the standard protocol, including a baseline and 3 follow-up surveys. During the visits to community clinics, standardized questionnaires were administrated by well-trained interviewers to collect information on personal characteristics, lifestyle, medical history, and anthropometric measurements with stringent quality control. Education level, smoking, alcohol and diet consumption, and physical activity were self-reported by participants. Participants who smoked were those who smoked 400 cigarettes or more or 500 g or more of tobacco leaves, or smoked at least 1 cigarette every day for a year. Individuals who drank alcohol were those who consumed alcohol at least once a week in the previous year. According to guidelines, a modified diet score for Chinese cardiovascular health was defined as the numbers of healthy components (consumption of ≥500 g/d of fruits and vegetables; ≥200 g/week of fish; ≥125 g/d of soybean products; <75 g/d of red meat; and ≥50 g/mo of tea).^26,27,28^ It was used in this study based on the evidence that the 5 components in the score were all related to blood lipids.^5,29,30^ Ideal physical activity was defined as having at least 150 minutes per week of moderate physical activity, at least 75 minutes per week of vigorous physical activity, or at least 150 minutes per week of both. Height and weight were measured, and body mass index (BMI) was calculated as weight in kilograms divided by height in meters squared. Ten-hour fasting blood samples were drawn from participants. Serum lipid levels, including total cholesterol (TC), TG, and high-density lipoprotein cholesterol (HDL-C), were measured in the laboratory which participated in the Lipid Standardization Program of the United States Centers for Disease Control and Prevention. LDL-C was calculated using the Friedewald formula.^31^

### Variant Selection, Genotyping, and PRS

We included 130 genetic variants from 3 large genome-wide association studies in East Asia.^18,32,33^ Subsequently, Illumina Hiseq X Ten sequencer was used to genotype participants’ samples by multiplex polymerase chain reaction targeted amplicon sequencing technology, with a 99.9% call rate and 990× median sequencing depth. We eliminated the correlated variants in high linkage disequilibrium using the pruning technique (r ^2^ >0.6) for each lipid trait, and 126 genetic variants remained for developing the PRSs of blood lipids (eTable 2 in Supplement 1). The PRS for each lipid trait was estimated as the sum of the number of risk alleles at each variant multiplied by the selected effect sizes according to previous studies.^18,33,34^

The PRSs for TC, TG, LDL-C, and HDL-C were categorized into 5 groups according to the quintiles among male and female participants, separately. The low, intermediate, and high polygenic risk were defined as the first, second to fourth, and fifth quintiles, respectively.

### Rate of Blood Lipid Change

The estimated annual changes (EACs) of lipid levels were used to reflect rates of blood lipid changes in each interval, which were calculated as the difference in blood lipid levels between any 2 adjacent examinations divided by their time interval (year). Positive and negative values represented increase and decrease of lipid levels, respectively.

### Statistical Analysis

Sex-specific baseline characteristics of study participants were expressed as means with standard deviations for continuous variables or numbers and percentages for categorical variables and were compared using a *t* test or χ^2^ test, respectively. The analytic data set included 37 317 participants without missing data and included EACs of blood lipids for each participant in each interval and risk factors at the beginning of each interval. Associations of EACs of blood lipids with age and polygenic risk were assessed using generalized estimating equations for repeated measures analyses with empirical estimates of standard errors. A term for participant cluster was included in all analyses accounting for nonindependence of participants with an unstructured correlation structure. Potential confounders were first chosen according to previous literature and determined by univariable analyses (*P* < .05). Multivariate analyses were then performed to adjust for these confounders at the beginning of 2 adjacent examinations, including region (Northern China or Southern China), area (urban or rural), subcohort, sex, age, education level (high school and above or less than high school), lipid level, smoking, alcohol consumption, BMI (<25 or ≥25), physical activity (ideal or nonideal), diet score (<2 or ≥2), and survey year. The linear trends were tested using the median age (or PRS) in each age (or PRS) category as a continuous variable in the generalized estimating equations. Interactions of polygenetic risk and age on blood lipid changes were examined by including an additional interaction term in the models. Sensitivity analysis was conducted by excluding participants with lipid treatment during the study period. All statistical analyses were performed using SAS 9.4 (SAS Institute). All tests were 2-sided, and *P* < .05 was considered statistically significant. Data were analyzed from June to August 2022.

## Results

### Characteristics of Study Participants

Among 37 317 participants with a mean (SD) age of 51.37 (10.82) years, 15 664 (41.98%) were male (Table 1). Male participants, compared with female participants, were more likely to live in urban areas (3564 [22.75%] vs 4003 [18.49%]), smoke (10 112 [64.64%] vs 651 [3.02%]), drink alcohol (6532 [41.75%] vs 919 [4.25%]). Male participants had higher education levels (3733 [23.96%] vs 3090 [14.37%]), diet scores (11 461 [74.18%] vs 14 209 [66.67%], and a higher prevalence of ideal physical activity (10 291 [66.43%] vs 12 875 ([60.89%]) and lower BMI (23.56 [3.44] vs 24.15 [3.75]), TC (178.55 [35.99] vs 182.27 [36.35] mg/dL), natural log-transformed TG (4.79 [0.56] vs 4.80 [0.54]), LDL-C (101.16 [31.33] vs 102.72 [31.61] mg/dL), and HDL-C (50.11 [13.83] vs 52.08 [12.72] mg/dL) levels.

**Table 1.** Baseline Characteristics of the Study Participants Stratified by Sex^a^

Variable	Participants, No. (%)		*P* value
	Male (n = 15 664)	Female (n = 21 653)	
Age, mean (SD), y	51.57 (10.87)	51.22 (10.78)	.002
North China	7921 (50.57)	10 822 (49.98)	.26
Urban	3564 (22.75)	4003 (18.49)	<.001
High school education and above	3733 (23.96)	3090 (14.37)	<.001
Smoking	10 112 (64.64)	651 (3.02)	<.001
Alcohol drinking	6532 (41.75)	919 (4.25)	<.001
Ideal physical activity	10 291 (66.43)	12 875 (60.89)	<.001
Diet score ≥2	11 461 (74.18)	14 209 (66.67)	<.001
BMI, mean (SD)	23.56 (3.44)	24.15 (3.75)	<.001
TC, mean (SD), mg/dL	178.55 (35.99)	182.27 (36.35)	<.001
Natural log-transformed TG, mean (SD)	4.79 (0.56)	4.80 (0.54)	.01
LDL-C, mean (SD), mg/dL	101.16 (31.33)	102.72 (31.61)	<.001
HDL-C, mean (SD), mg/dL	50.11 (13.83)	52.08 (12.72)	<.001

Abbreviations: BMI, body mass index (calculated as weight in kilograms divided by height in meters squared); HDL-C, high-density lipoprotein cholesterol; LDL-C, low-density lipoprotein cholesterol; TC, total cholesterol; TG, triglyceride.

SI conversion factor: To convert TC, LDL-C, and HDL-C to mmol/L, multiply by 0.0259.

^a^ Values are expressed as mean (SD) or No. (%) and were compared using *t* test or χ^2^ test, respectively.

We also compared sex-specific lipid levels at the beginning of any 2 adjacent examinations. Female participants had lower TC, TG, and LDL-C level than male participants before the age of 50 years, but opposite findings emerged afterward (eFigure 2 in Supplement 1). Female participants had higher HDL-C level than male participants across all age groups.

### Associations of EACs With Age

**Figure 1.**
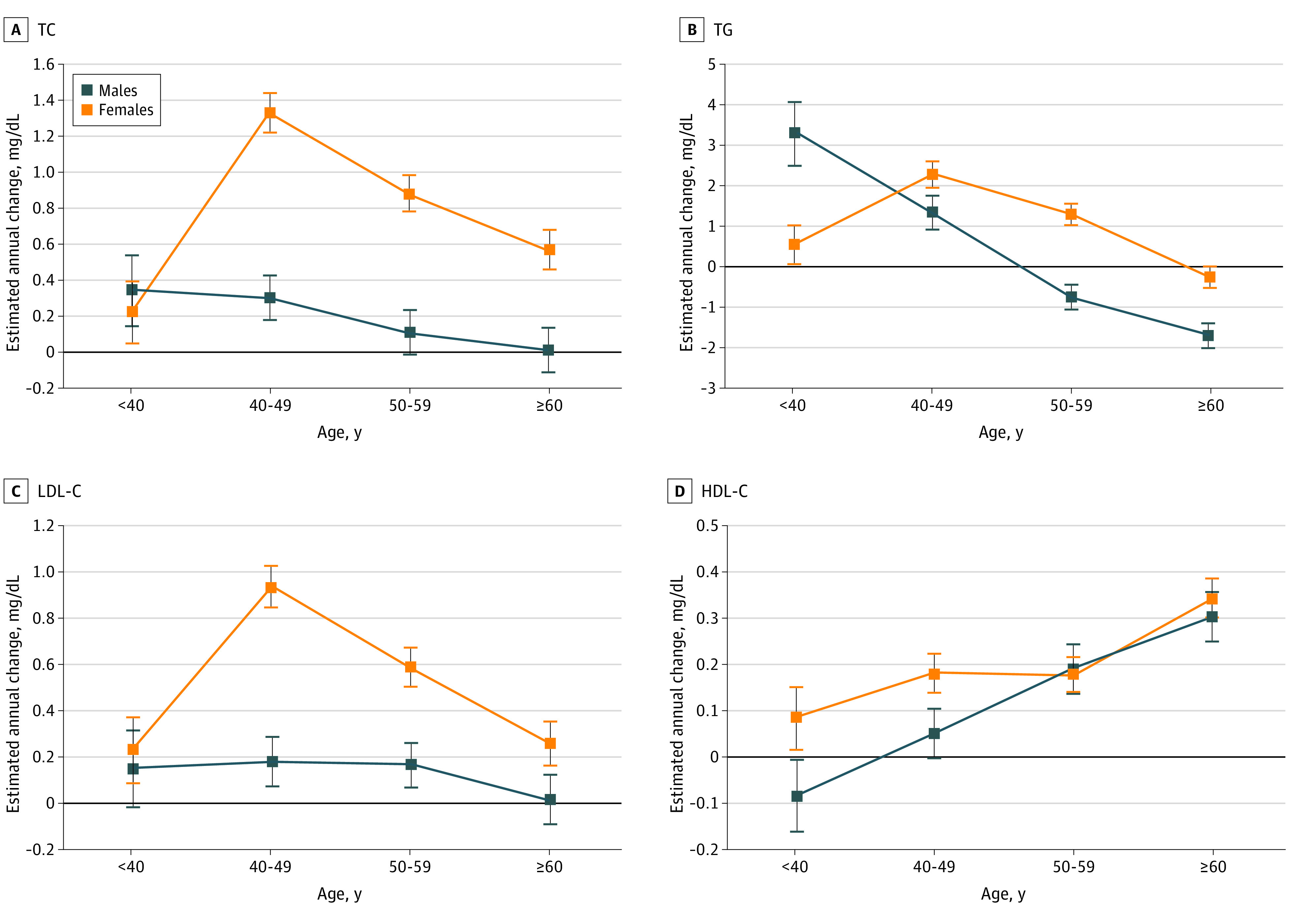
Multivariable-Adjusted Estimated Annual Changes of Lipid by Age Group Results were adjusted for region, area, subcohort, education level, lipid level, smoking, alcohol consumption, body mass index (calculated as weight in kilograms divided by height in meters squared), physical activity, diet, and survey year at the beginning of 2 adjacent examinations. Squares indicate the estimated annual changes of lipid, and error bars represent the 95% CIs. SI conversion factor: To convert TC, LDL-C, and HDL-C to mmol/L, multiply by 0.0259; to convert TG to mmol/L, multiply by 0.0113. HDL-C indicates high-density lipoprotein cholesterol; LDL-C, low-density lipoprotein cholesterol; TC, total cholesterol; TG, triglyceride.

We estimated the associations between age and EACs of blood lipids (Figure 1 and eTable 3 in Supplement 1). There were different association patterns between male and female participants. Male participants experienced declining change as they got older for TC (EAC, 0.34 [95% CI, 0.14 to 0.54] mg/dL for age <40 years vs 0.01 [95% CI, −0.11 to 0.13] mg/dL for age ≥60 years), TG (EAC, 3.28 [95% CI, 2.50 to 4.07] mg/dL for age <40 years vs −1.70 [95% CI, −2.02 to −1.38] mg/dL for age ≥60 years), and LDL-C (EAC, 0.15 [95% CI, −0.02 to 0.32] for age <40 years vs 0.01 [95% CI, −0.10 to 0.11] mg/dL for age ≥60 years) (all *P* for trend <.05). However, we found inverse V-shaped associations between age and EACs of TC, TG, and LDL-C among female participants, with the greatest rate of change appearng in the age group of 40 to 49 years (EAC for TC, 1.33 [95% CI, 1.22 to 1.44] mg/dL; EAC for TG, 2.28 [95% CI, 1.94 to 2.62] mg/dL; and EAC for LDL-C, 0.94 [95% CI, 0.84 to 1.03] mg/dL). EAC of HDL-C among female participants increased significantly after the age of 60. Female participants had higher EACs of TC, TG, and LDL-C than male participants over 40 years.

### Associations of EACs With Polygenic Risk

The associations of polygenic risk with EACs of blood lipids were evaluated. Rates of change for TC, TG, LDL-C, and HDL-C increased significantly with elevated polygenic risk in both sexes (all *P* for trend <.001) (Table 2; eFigure 3 in Supplement 1). Notably, blood lipid levels among participants at low polygenic risk tended to shift toward lower levels, with EACs of −0.16 (95% CI, −0.25 to −0.07) mg/dL, −1.58 (95% CI, −1.78 to −1.37) mg/dL, −0.13 (95% CI, −0.21 to −0.06) mg/dL, and −0.11 (95% CI,−0.15 to −0.08) mg/dL for TC, TG, LDL-C, and HDL-C, respectively. These decreasing patterns of EACs were more pronounced in male participants at low polygenic risk than those in low-risk female participants. For low-risk male participants, EACs of LDL-C were −0.33 (95% CI, −0.44 to −0.22) mg/dL, while EACs were 0.02 (95% CI, −0.07 to 0.12) mg/dL for low-risk female participants. Conversely, individuals at high polygenic risk showed the greatest EACs (TC: 1.12 [95% CI, 1.03 to 1.21] mg/dL; TG: 3.57 [95% CI, 3.24 to 3.91] mg/dL; LDL-C, 0.73 [95% CI, 0.65 to 0.81] mg/dL; and HDL-C, 0.51 [95% CI, 0.48 to 0.55] mg/dL) (Table 2).

**Table 2.** Multivariable-Adjusted Estimated Annual Changes of Lipid in Milligrams per Deciliter and 95% CIs According to Polygenic Risk Group^a^

Blood lipid	Polygenic risk			*P* value for trend
	Low	Intermediate	High	
**Total**				
TC	−0.16 (−0.25 to −0.07)	0.60 (0.55 to 0.66)	1.12 (1.03 to 1.21)	<.001
TG	−1.58 (−1.78 to −1.37)	0.22 (0.07 to 0.36)	3.57 (3.24 to 3.91)	<.001
LDL-C	−0.13 (−0.21 to −0.06)	0.42 (0.37 to 0.46)	0.73 (0.65 to 0.81)	<.001
HDL-C	−0.11 (−0.15 to −0.08)	0.20 (0.18 to 0.22)	0.51 (0.48 to 0.55)	<.001
**Male**				
TC	−0.59 (−0.72 to −0.46)	0.21 (0.14 to 0.29)	0.73 (0.60 to 0.87)	<.001
TG	−2.12 (−2.45 to −1.79)	−0.45 (−0.68 to −0.23)	2.89 (2.37 to 3.41)	<.001
LDL-C	−0.33 (−0.44 to −0.22)	0.14 (0.07 to 0.21)	0.51 (0.39 to 0.64)	<.001
HDL-C	−0.14 (−0.19 to −0.08)	0.16 (0.12 to 0.19)	0.48 (0.42 to 0.54)	<.001
**Female**				
TC	0.17 (0.05 to 0.29)	0.91 (0.84 to 0.97)	1.42 (1.29 to 1.54)	<.001
TG	−1.17 (−1.44 to −0.90)	0.74 (0.55 to 0.93)	4.18 (3.74 to 4.62)	<.001
LDL-C	0.02 (−0.07 to 0.12)	0.63 (0.57 to 0.69)	0.89 (0.78 to 1.00)	<.001
HDL-C	−0.09 (−0.14 to −0.05)	0.22 (0.20 to 0.25)	0.53 (0.49 to 0.58)	<.001

Abbreviations: HDL-C, high-density lipoprotein cholesterol; LDL-C, low-density lipoprotein cholesterol; TC, total cholesterol; TG, triglyceride.

SI conversion factor: To convert TC, LDL-C, and HDL-C to mmol/L, multiply by 0.0259; to convert TG to mmol/L, multiply by 0.0113.

^a^ Adjusted for sex (only for total population), region, area, subcohort, age, education level, lipid level, smoking, alcohol consumption, body mass index (calculated as weight in kilograms divided by height in meters squared), physical activity, diet, and survey year at the beginning of 2 adjacent examinations. The low, intermediate, and high polygenic risk were defined as the first, second to fourth, and fifth quintiles of polygenic risk scores.

### Associations of EACs With Combined Age and Polygenic Risk

**Figure 2.**
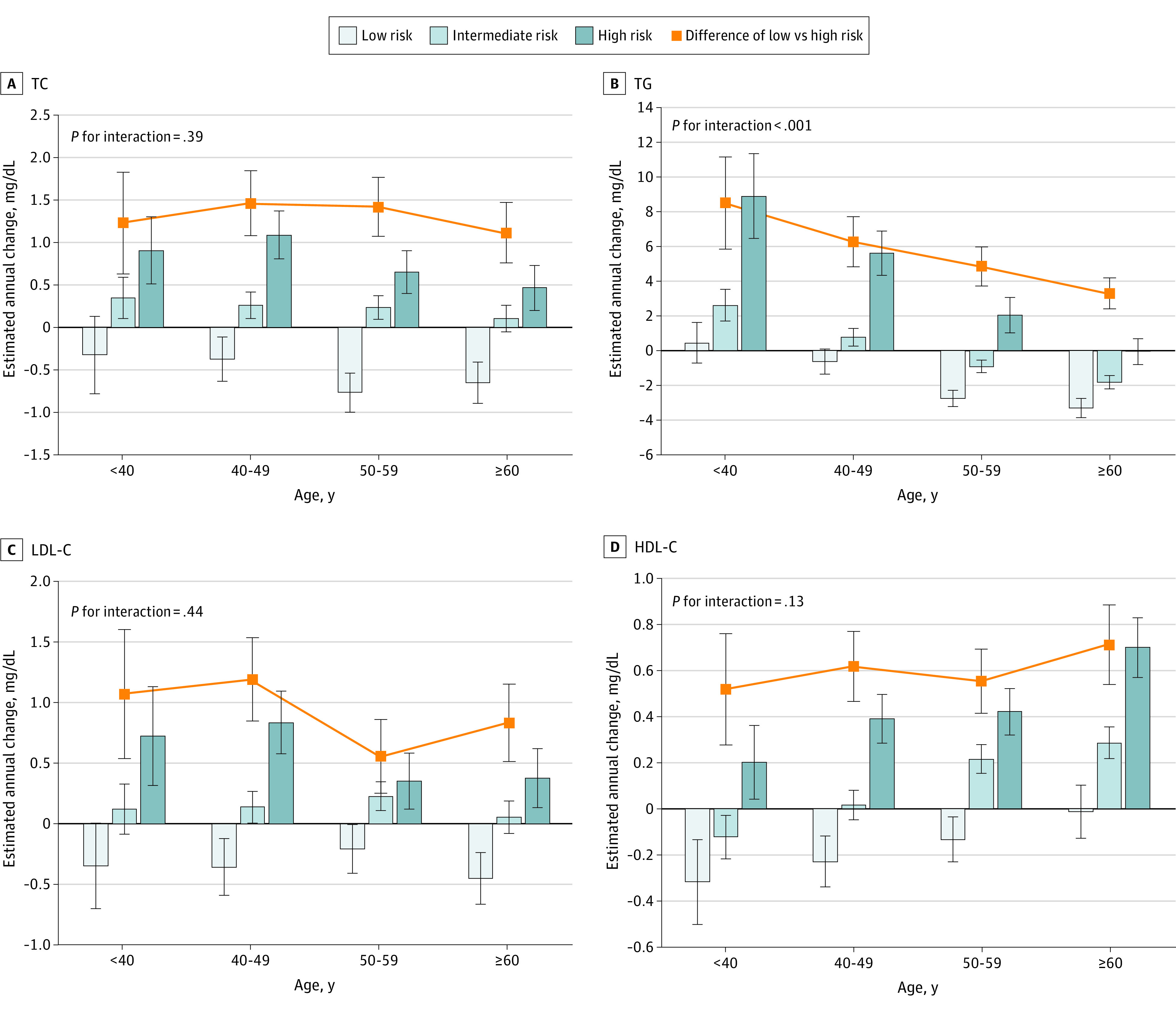
Multivariable-Adjusted Estimated Annual Changes of Lipid According to Polygenic Risk and Age Group Among Male Participants Results were adjusted for region, area, subcohort, education level, lipid level, smoking, alcohol consumption, body mass index (calculated as weight in kilograms divided by height in meters squared), physical activity, diet, and survey year at the beginning of 2 adjacent examinations. The low, intermediate, and high polygenic risk were defined as the first, second to fourth, and fifth quintiles of polygenic risk scores. Bars are the estimated annual changes, squares are differences of the estimated annual changes between low and high polygenic risk, and error bars represent the 95% CIs. SI conversion factor: To convert TC, LDL-C, and HDL-C to mmol/L, multiply by 0.0259; to convert TG to mmol/L, multiply by 0.0113. HDL-C indicates high-density lipoprotein cholesterol; LDL-C, low-density lipoprotein cholesterol; TC, total cholesterol; TG, triglyceride.

**Figure 3.**
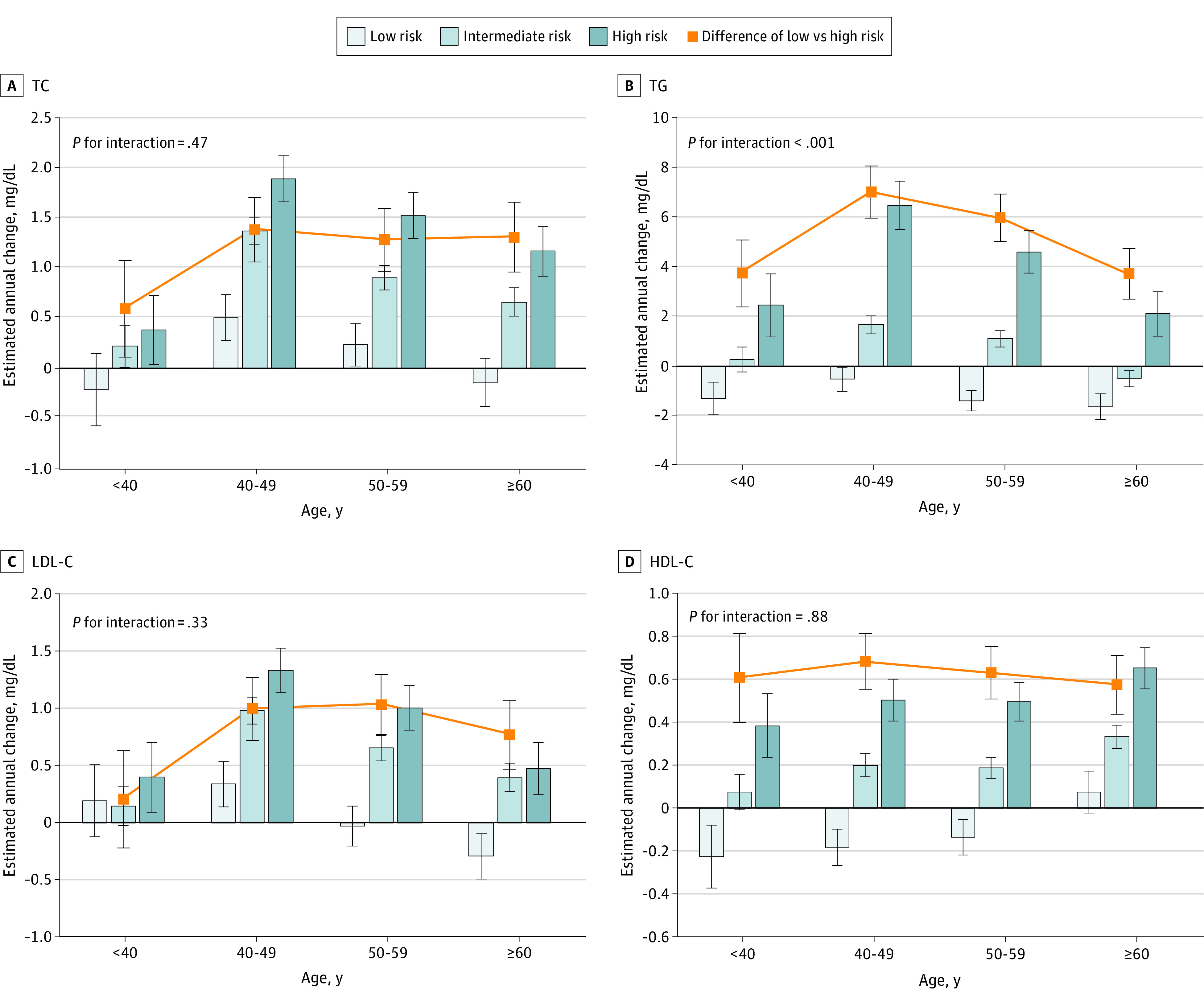
Multivariable-Adjusted Estimated Annual Changes of Lipid According to Polygenic Risk and Age Group Among Female Participants Results were adjusted for region, area, subcohort, education level, lipid level, smoking, alcohol consumption, body mass index (calculated as weight in kilograms divided by height in meters squared), physical activity, diet, and survey year at the beginning of 2 adjacent examinations. The low, intermediate, and high polygenic risk were defined as the first, second to fourth, and fifth quintiles of polygenic risk scores. Bars are the estimated annual changes, squares are differences of the estimated annual changes between low and high polygenic risk, and error bars represent the 95% CIs. SI conversion factor: To convert TC, LDL-C, and HDL-C to mmol/L, multiply by 0.0259; to convert TG to mmol/L, multiply by 0.0113. HDL-C indicates high-density lipoprotein cholesterol; LDL-C, low-density lipoprotein cholesterol; TC, total cholesterol; TG, triglyceride.

The associations of EACs with age and polygenic risk were explored with a multivariable-adjusted analysis (Figure 2 and Figure 3). Positive genetic associations with EACs of 4 lipid indicators were found across sex and age groups. Lipid levels among participants at low polygenic risk tended to fall gradually or remain steady in each age group, particularly among male participants. EACs of LDL-C among low-risk male participants younger than 40 years were −0.35 (95% CI, −0.70 to 0.01) mg/dL; aged 40 to 49 years, −0.36 (95% CI, −0.59 to −0.12) mg/dL; aged 50 to 59 years, −0.21 (95% CI, −0.41 to −0.01) mg/dL; and aged 60 years or older, −0.45 (95% CI, −0.66 to −0.24) mg/dL. Conversely, participants at high polygenic risk had the greatest EACs toward higher lipid levels across all age groups. It is worth noting that the genetic association with EAC of TG was modified by age (*P* for interaction <.001 for both male and female participants). The differences in EAC of TG between low and high polygenic risk groups decreased from 8.47 (95% CI, 5.81 to 11.13) mg/dL among male participants younger than 40 years to 3.27 (95% CI, 2.37 to 4.16) mg/dL among those aged 60 years or older, and EACs declined from 7.00 (95% CI, 5.96 to 8.05) mg/dL among female participants aged 40 to 49 years to 3.73 (95% CI, 2.70 to 4.75) mg/dL among those aged 60 years or older. No interaction between genetic risk and age was observed for EACs of TC, LDL-C, and HDL-C.

### Sensitivity Analysis

To avoid the influence of lipid treatment on blood lipid changes, we excluded all participants with lipid-lowering therapy during the study period and conducted a sensitivity analysis. It demonstrated similar results to the main analysis (eTable 4 and eFigure 4-6 in Supplement 1).

## Discussion

Using a large-scale population-based longitudinal cohort with repeated measurements, we assessed the associations of blood lipid changes with age and polygenic risk. Our results suggested that EACs of TC, TG, and LDL-C among male participants decreased with age, whereas female participants had inverse V-shaped associations with peak EACs at age 40 to 49 years. The polygenic risk was also associated with the directions and rates of lipid changes, which was observed in both sexes and all age groups. Several significant findings of this study could have relevant clinical implications.

First, we examined the associations of lipid EACs with age. It is well known that blood lipid levels tend to increase with age,^12,13,14^ but it is unclear whether and to what extent lipid change rates could vary with age. Our study demonstrated that the EACs of TC, TG, and LDL-C declined gradually with age in male participants, and fell sharply in female participants after the age of 40 years, which might be attributed to age-related changes of basal metabolism, the liver sinusoidal endothelium, postprandial lipemia, insulin resistance, growth hormone, and peroxisome proliferator-activated receptor α activity.^35^ It is suggested that lipid interventions could be implemented in the critical age window (before age 40 years for male participants and 40 to 49 years for female participants) when lipid levels deteriorated at the relatively greater rate than others. Guidelines on lipid management are always aimed at population with higher lipid levels,^1,7^ but younger individuals are more likely to have lower lipid levels, which conceals the fact that they have the greater EACs toward unfavorable lipid profiles and required intensive lifestyle interventions. Consequently, we propose that lipid control should focus more on the critical age window with a higher rate of deterioration, which will yield more health benefits.

Second, we identified sex differences in the associations between rates of lipid changes and age. For EACs of TC, TG, and LDL-C with age, there were decreasing trends among male participants, but inverse V-shaped associations among female participants. The similar inverse V-shaped associations among female participants could also be deduced from a Chinese study with nationally representative sample, using differences in mean lipid levels between any 2 adjacent age groups.^14^ The significant increases of EACs among female participants at age group of 40 to 49 could explain why blood lipid levels among female participants subsequently rose rapidly,^13,14^ and were consistent with a substantial rise in prevalence of abdominal obesity.^36,37^ It might be due to the decrease in estrogen among female participants during perimenopause and menopause. Another potential explanation is that their lifestyles tend to change toward overnutrition and physical inactivity during and after pregnancy.^38^ Interestingly, female participants had higher lipid levels and EACs than male participants beyond the age of 40 years, suggesting that more attention should be paid to lipid management in female participants, especially those who were perimenopausal and menopausal.

Third, we found that genetic risk was associated with the directions and rates of changing blood lipids. Although several studies have reported that PRSs were predictive of longitudinal lipid changes, there is limited evidence in China.^39,40^ A previous study on the Chinese population identified linear associations between the lipid changes and PRSs, which included 20 genetic variants.^32^ In the current study, we generated the PRSs using 126 significant genetic variants for lipid traits in the East Asian population,^18,32,33^ further expanded to a larger sample size with repeated lipid measurements, and comprehensively estimated the associations of PRSs with lipid changes, which showed similar results with previous studies. Our study further indicated that lipid levels among individuals at low polygenic risk tended to decrease or remain stable, whereas lipid levels among high-risk individuals accelerated toward unhealthy profiles at high rates. Specifically, people at the highest 20% of genetic risk should be paid more attention to maximize potential health benefits from management of cholesterol and triglycerides. Polygenic risk assessment could motivate those at high risk to adhere to healthy lifestyle modifications,^23,41^ especially when considering that the treatment and control of dyslipidemia was extremely low in China.^14^ Moreover, PRS could increase the accuracy of risk estimation for individuals with familial hypercholesterolemia.^42^ The clinical benefits of polygenic risk should be confirmed by randomized controlled trials.

This study has several strengths. First, we estimated the associations of blood lipid changes with age and polygenic risk using a large-scale population-based prospective longitudinal cohort with 4 repeated measurements over a mean follow-up period of 13.8 years. Second, our findings were reliable due to the well-defined phenotypes and rigorous quality control. Third, available repeated measurements of multiple risk factors in our study enable us to better control for potential confounders.

### Limitations

Some potential limitations should also be noted. First, stratification based on age and PRS resulted in relatively small sample sizes for some groups, especially among younger individuals at low or high genetic risk. It might decrease statistical power and cause some uncertainties. Second, blood lipids could be influenced by other lifestyle factors not captured in this study, which might affect the associations. But confounding should be limited, given that we have adjusted for major lifestyle factors in the analysis. Third, self-reported confounders, such as smoking, physical activity and diet, might be associated with our results due to recall bias. Fourth, missing data might induce selection bias, while their impact should be slight due to similar characteristics between the included and excluded samples. Finally, our study was conducted in Chinese population, generalization to other ethnicities should be done with caution.

## Conclusions

The findings of this cohort study suggests that lipid change rates are associated with age and genetic predisposition in Chinese adults. Therefore, precision prevention strategies for blood lipids should be optimized by taking account of both genetic risk and critical age window, which might offer an opportunity to reduce unhealthy lipid traits and subsequent cardiovascular burden.

## Notes

Supplement 1.
eMethods.
**eTable 1.** Baseline characteristics for the included and excluded participants **eTable 2.** Genetic variants selected in this study **eTable 3.** Sample sizes according to sex, age, and polygenic risk group **eTable 4.** Multivariable-adjusted estimated annual changes of lipid (mg/dL) and 95% CIs according to polygenic risk group among participants without lipid treatment **eFigure 1.** Flow chart of study participants included and excluded in the analysis **eFigure 2.** Blood lipid levels at the beginning of any 2 adjacent examinations **eFigure 3.** Multivariable-adjusted estimated annual changes of lipid by quintiles of polygenic risk **eFigure 4.** Multivariable-adjusted estimated annual changes of lipid by age group among participants without lipid treatment **eFigure 5.** Multivariable-adjusted estimated annual changes of lipid according to polygenic risk and age group among male participants without lipid treatment **eFigure 6.** Multivariable-adjusted estimated annual changes of lipid according to polygenic risk and age group among female participants without lipid treatment

Supplement 2.
Data Sharing Statement
