# Supplementary material for: Age and Genetic Risk Score and Rates of Blood Lipid Changes in China
Source: JAMA Netw Open. 2023 Mar 29;6(3):e235565. doi: 10.1001/jamanetworkopen.2023.5565 (PMC10061238; doi:10.1001/jamanetworkopen.2023.5565)
Supplement: Supplement 1. — eMethods. eTable 1. Baseline characteristics for the included and excluded participants eTable 2. Genetic variants selected in this study eTable 3. Sample sizes according to sex, age, and polygenic risk group eTable 4. Multivariable-adjusted estimated annual changes of lipid (mg/dL) and 95% CIs according to polygenic risk group among participants without lipid treatment eFigure 1. Flow chart of study participants included and excluded in the analysis eFigure 2. Blood lipid levels at the beginning of any 2 adjacent examinations eFigure 3. Multivariable-adjusted estimated annual changes of lipid by quintiles of polygenic risk eFigure 4. Multivariable-adjusted estimated annual changes of lipid by age group among participants without lipid treatment eFigure 5. Multivariable-adjusted estimated annual changes of lipid according to polygenic risk and age group among male participants without lipid treatment eFigure 6. Multivariable-adjusted estimated annual changes of lipid according to polygenic risk and age group among female participants without lipid treatment [file jamanetwopen-e235565-s001.pdf]

Supplemental Online Content

Li J, Liu M, Liu F, et al. Age and genetic risk score and rates of blood lipid changes in China. *JAMA Netw Open*. 2023;6(3):e235565. doi:10.1001/jamanetworkopen.2023.5565

eMethods.
eTable 1. Baseline characteristics for the included and excluded participants
eTable 2. Genetic variants selected in this study
eTable 3. Sample sizes according to sex, age, and polygenic risk group
eTable 4. Multivariable-adjusted estimated annual changes of lipid (mg/dL) and 95% CIs according to polygenic risk group among participants without lipid treatment
eFigure 1. Flow chart of study participants included and excluded in the analysis
eFigure 2. Blood lipid levels at the beginning of any 2 adjacent examinations
eFigure 3. Multivariable-adjusted estimated annual changes of lipid by quintiles of polygenic risk
eFigure 4. Multivariable-adjusted estimated annual changes of lipid by age group among participants without lipid treatment
eFigure 5. Multivariable-adjusted estimated annual changes of lipid according to polygenic risk and age group among male participants without lipid treatment
eFigure 6. Multivariable-adjusted estimated annual changes of lipid according to polygenic risk and age group among female participants without lipid treatment

This supplemental material has been provided by the authors to give readers additional information about their work.

eMethods.

Detailed description of this study

Study cohort

The China Multi-Center Collaborative Study of Cardiovascular Epidemiology (China MUCA) was conducted in 1998, and recruited 11 480 participants aged 35-59 years from 11 clusters by the cluster random sampling method, with about 1000 participants in each cluster. The International Collaborative Study of Cardiovascular Disease in Asia (InterASIA) was established in 2000-2001, and included 15 540 participants aged 35-74 years from 10 Chinese provinces using the four-stage stratified sampling method. The Community Intervention of Metabolic Syndrome in China and Chinese Family Health Study (CIMIC) was established in 2007-2008, and enrolled 86 428 participants aged 18 years or older from four survey sites in three Chinese provinces, with approximately 20 000 participants in each survey site. There were 8131 participants in the China MUCA and 13 862 participants in the InterASIA with available genotype data. Using the stratified sampling method by survey site, a subset of 25 698 individuals was selected from the CIMIC for genotyping. Therefore, this study included 47 691 participants with available genotype data. Information of them was combined using unified definitions of risk factors under a standard protocol.

According to a uniform protocol, the InterASIA and ChinaMUCA were firstly followed up to collect information on blood lipid levels and the related risk factors during 2007-2008. The second and third follow-up surveys were conducted for all three subcohorts using a uniform protocol during 2012-2015 and 2018-2020, respectively.

Quality control

All interviewers and staffs in this study successfully completed the training program, which introduced them to the aims of the study and the specific methodologies and tools used. They were given detailed instructions on how to administer the study questionnaires and measurements at the training sessions. The qualified interviewers collected information of participants using standardized questionnaires and tools in accordance with a standard protocol under stringent quality control. Blood specimens were drawn and processed at the examination center. Blood lipids were subsequently measured in study laboratories according to the criteria of the Lipid Standardization Program of the United States Centers for Disease Control and Prevention.

A Data Monitoring and Management Committee was established to provide comprehensive monitoring for this study, assess the accuracy and reliability of examination methods and control variability, including collection of quality control data. When reliability or variability is unacceptable, the committee will alert the examination centers, and oversee further investigation and corrective action, as appropriate.

Sample size calculation

Sample size calculation was conducted using the “longpower” package in R software which supported models fitted by generalized estimating equations. We calculated sample sizes for high-density lipoprotein cholesterol, which theoretically required a larger sample size to draw credible conclusions due to its expected smaller associations with age and polygenic risk. The estimated sample size is 10 222 for males and 10 417 for females, respectively, at α=0.05, effect size=0.1, and power=85%. Therefore, sample size in this study is large enough to support our conclusions.

© 2023 Li J et al. *JAMA Network Open.*

eTable 1. Baseline Characteristics for the Included and Excluded Participants

Variable	Included participants	Excluded participants[a]
N	37 317	6662
Male, n (%)	15 664 (41.98)	3265 (49.01)
Age, year	51.37±10.82	56.29±13.96
North China, n (%)	18 743 (50.23)	2516 (37.77)
Urban, n (%)	7567 (20.28)	1260 (18.91)
High school education and above, n (%)	6823 (18.40)	1203 (18.12)
Smoking, n (%)	10 763 (28.92)	2127 (32.02)
Alcohol drinking, n (%)	7451 (19.99)	1348 (20.24)
Physical activity (ideal), n (%)	23 166 (63.24)	3728 (56.09)
Diet score (≥2), n (%)	25 670 (69.82)	4437 (67.23)
Body mass index, kg/m^2	23.90±3.63	23.29±3.78
TC, mg/dL	180.71±36.25	176.10±37.49
ln (TG)	4.80±0.55	4.78±0.53
LDL-C, mg/dL	102.07±31.50	98.41±32.36
HDL-C, mg/dL	51.25±13.24	50.94±13.38

Abbreviations: TC, total cholesterol; TG, triglyceride; LDL-C, low-density lipoprotein cholesterol; HDL-C, high-density lipoprotein cholesterol; ln, natural log-transformed.

Values are expressed as mean ± standard deviation or numbers (percentage).

a, participants were excluded due to unavailable blood lipid data at any follow-up survey

SI conversion factor: To convert TC, LDL-C, and HDL-C to mmol/L, multiply by 0.0259; to convert TG to mmol/L, multiply by 0.0113.

© 2023 Li J et al. *JAMA Network Open.*

eTable 2. Genetic Variants Selected in This Study

Genotyped variants	Chr: Pos_hg19	Initially selected variants	Gene	Effect allele	Other allele	Effect size[a]	MAF in cohort	Reference for variant selection
TC								
rs1077834	15:58723479	rs1077834	LIPC	C	T	0.05941	0.3966	Ref 2
rs10889353	1:63118196	rs10889353	DOCK7	C	A	-0.05622	0.1952	Ref 3
rs11136341	8:145043543	rs11136341	PLEC	A	G	-0.0418	0.1087	Ref 2
rs1129555	10:113910721	rs1129555	GPAM	G	A	-0.02603	0.3006	Ref 2
rs11557092	19:11257018	rs11557092	SPC24	C	T	0.05627	0.2574	Ref 3
rs1169288	12:121416650	rs1169288	HNF1A	C	A	0.0379816	0.4062	Ref 3
rs117711462	1:93159927	rs117711462	EVI5	A	G	0.2115	0.006603	Ref 3
rs12027135	1:25775733	rs12027135	TMEM57	A	T	-0.0292	0.2939	Ref 2
rs12042319	1:63049819	rs12042319	DOCK7	A	G	-0.03266	0.1957	Ref 1
rs12453914	17:67138878	rs12453914	ABCA6	A	C	0.01427	0.4431	Ref 1
rs1260326	2:27730940	rs1260326	GCKR	C	T	-0.03525	0.489	Ref 3
rs12740374	1:109817590	rs12740374	CELSR2	T	G	-0.08065	0.0599	Ref 3
rs12927205	16:72025077	rs12927205	PKD1L3	A	G	0.0666	0.2632	Ref 1
rs13277801	8:59353534	rs13277801	UBXN2B	T	C	-0.02019	0.2025	Ref 2
rs13306194	2:21252534	rs13306194	APOB	A	G	-0.07659	0.1193	Ref 3
rs1367117	2:21263900	rs1367117	APOB	A	G	0.05277	0.1271	Ref 3
rs1495741	8:18272881	rs1495741	NAT2	A	G	-0.01817	0.4538	Ref 3
rs151193009	1:55509585	rs151193009	PCSK9	T	C	-0.5081294	0.01422	Ref 3
rs1532085	15:58683366	rs1532085		G	A	-0.04192	0.4596	Ref 3
rs17122278	11:118449370	rs17122278	ARCN1	A	G	-0.0469	0.4996	Ref 2
rs17358402	16:71967927	rs17358402	PKD1L3	T	C	0.088	0.05423	Ref 3
rs174546	11:61569830	rs174546	FADS1	T	C	-0.03215	0.4576	Ref 2
rs174547	11:61570783	rs174547	FADS1	C	T	-0.03215	0.4578	Ref 3
rs1800588	15:58723675	rs1800588	LIPC	T	C	0.05965	0.3788	Ref 3
rs1883025	9:107664301	rs1883025	ABCA1	T	C	-0.05607	0.222	Ref 3
rs2000999	16:72108093	rs2000999	HPR	A	G	0.04394	0.2594	Ref 3
rs200990725	19:11217315	rs200990725	LDLR	T	C	0.6771	0.001208	Ref 3
rs2043085	15:58680954	rs2043085	ALDH1A2	C	T	-0.0398	0.4645	Ref 3
rs2066714	9:107586753	rs2066714	ABCA1	C	T	0.03453	0.2808	Ref 3
rs2081687	8:59388565	rs2081687	CYP7A1	C	T	-0.04142	0.211	Ref 3
rs2230808	9:107562804	rs2230808	ABCA1	C	T	0.02249	0.38	Ref 3
rs2297991	10:113913222	rs2297991	GPAM	C	T	-0.0432414	0.301	Ref 3
rs247616	16:56989590	rs247616	AC012181.1	T	C	0.05349	0.163	Ref 3
rs2575876	9:107665739	rs2575876	ABCA1	A	G	-0.05618	0.2192	Ref 1
rs2642442	1:220973563	rs2642442		T	C	0.0549	0.1699	Ref 2
rs312949	2:21334283	rs312949	TDRD15	C	G	0.0351	0.277	Ref 1
rs3846663	5:74655726	rs3846663	HMGCR	T	C	0.04768	0.4702	Ref 3
rs4377290	2:158437683	rs4377290	ACVR1C	C	T	-0.0386	0.316	Ref 3
rs439401	19:45414451	rs439401	APOC1	C	T	-0.02173	0.4191	Ref 3

© 2023 Li J et al. *JAMA Network Open.*

Genotyped variants	Chr: Pos_hg19	Initially selected variants	Gene	Effect allele	Other allele	Effect size[a]	MAF in cohort	Reference for variant selection
rs4883201	12:9082581	rs4883201	PHC1	G	A	-0.0245	0.3201	Ref 2
rs4939883	18:47167214	rs4939883	SMUG1P1	C	T	0.03065	0.1889	Ref 3
rs507666	9:136149399	rs507666	ABO	A	G	0.04758	0.2075	Ref 3
rs579459	9:136154168	rs579459	ABO	C	T	0.04714	0.2072	Ref 2
rs58542926	19:19379549	rs58542926	TM6SF2	T	C	-0.06629	0.07182	Ref 3
rs651821	11:116662579	rs651821	APOA5	T	C	-0.02331	0.2742	Ref 3
rs6871667	5:74604742	rs6871667	JMY	A	G	0.04675	0.4422	Ref 1
rs6882076	5:156390297	rs6882076	TIMD4	C	T	0.04544	0.2672	Ref 3
rs7185272	16:72013797	rs7185272	PKD1L3	C	G	0.05364	0.256	Ref 3
rs7258950	19:11250139	rs7258950	SPC24	G	A	0.05438	0.2181	Ref 1
rs72654473	19:45414399	rs445925	APOC1	A	C	-0.281	0.08517	Ref 3
rs7306523	12:53393964	rs7306523	EIF4B	G	A	-0.01847	0.3035	Ref 3
rs737337	19:11347493	rs737337	DOCK6	C	T	-0.04973	0.2733	Ref 3
rs7525649	1:55499156	rs7525649	PCSK9	T	C	0.03556	0.349	Ref 1
rs7616006	3:12267648	rs7616006	LINC00690	G	A	-0.01501	0.3312	Ref 2
rs769449	19:45410002	rs769449	APOE	A	G	0.09834	0.08447	Ref 3
rs7770628	6:161018174	rs7770628	LPA	T	C	-0.0691525	0.1133	Ref 3
rs7965082	12:100800193	rs7965082	SLC17A8	T	C	-0.02394	0.4124	Ref 3
rs9357121	6:31240479	rs9357121	HLA-B	T	G	0.0513	0.171	Ref 1
rs9376090	6:135411228	rs9376090	HBS1L	T	C	0.0575	0.2775	Ref 2
rs9390698	6:101296389	rs9390698	ASCC3	A	G	0.01711	0.2708	Ref 3
TG								
rs10096633	8:19830921	rs10096633	LPL	T	C	-0.1697	0.09916	Ref 3
rs1037814	4:88049850	rs1037814	AFF1	C	T	0.02776	0.4182	Ref 2
rs10889353	1:63118196	rs10889353	DOCK7	C	A	-0.0719	0.1952	Ref 3
rs12042319	1:63049819	rs12042319	DOCK7	A	G	-0.07122	0.1957	Ref 1
rs1260326	2:27730940	rs1260326	GCKR	C	T	-0.08881	0.489	Ref 3
rs130071	6:31116210	rs130071	CCHCR1	A	G	0.06836	0.07801	Ref 3
rs13306194	2:21252534	rs13306194	APOB	A	G	-0.07323	0.1193	Ref 3
rs1495741	8:18272881	rs1495741	NAT2	A	G	-0.05324	0.4538	Ref 3
rs1532085	15:58683366	rs1532085		G	A	-0.0447	0.4596	Ref 3
rs157582	19:45396219	rs157582	TOMM40	T	C	0.04522	0.1912	Ref 1
rs16990971	20:44601293	rs16990971	ZNF335	G	A	0.06814	0.06137	Ref 3
rs17145738	7:72982874	rs17145738	TBL2	T	C	-0.1013	0.1093	Ref 2
rs174546	11:61569830	rs174546	FADS1	T	C	0.03332	0.4576	Ref 2
rs174547	11:61570783	rs174547	FADS1	C	T	0.03327	0.4578	Ref 3
rs1800234	22:46615880	rs1800234	PPARA	C	T	-0.0944593	0.04777	Ref 3
rs1800588	15:58723675	rs1800588	LIPC	T	C	0.06402	0.3788	Ref 3
rs180327	11:116623659	rs180327	BUD13	T	C	-0.1106	0.3411	Ref 3
rs1832007	10:5254847	rs1832007	AKR1C4	G	A	-0.04274	0.1002	Ref 2
rs2043085	15:58680954	rs2043085	ALDH1A2	C	T	-0.04373	0.4645	Ref 3

© 2023 Li J et al. *JAMA Network Open.*

Genotyped variants	Chr: Pos_hg19	Initially selected variants	Gene	Effect allele	Other allele	Effect size[a]	MAF in cohort	Reference for variant selection
rs2068888	10:94839642	rs2068888	CYP26A1	A	G	-0.03429	0.1821	Ref 3
rs2075260	12:109696838	rs2075260	ACACB	A	G	0.04275	0.2649	Ref 3
rs2075291	11:116661392	rs2075291	APOA5	A	C	0.2927	0.05777	Ref 3
rs2081687	8:59388565	rs2081687	CYP7A1	C	T	-0.0285788	0.211	Ref 3
rs2144300	1:230294916	rs2144300	GALNT2	T	C	-0.02985	0.1862	Ref 3
rs3129853	6:32398648	rs3129853	TBC1D22B	A	G	0.04289	0.1562	Ref 3
rs35332062	7:73012042	rs35332062	MLXIPL	A	G	-0.1034	0.1103	Ref 3
rs439401	19:45414451	rs439401	APOC1	C	T	0.0687205	0.4191	Ref 3
rs4719841	7:25997536	rs4719841	MIR148A	G	A	0.02423	0.3913	Ref 3
rs58542926	19:19379549	rs58542926	TM6SF2	T	C	-0.06384	0.07182	Ref 3
rs651821	11:116662579	rs651821	APOA5	T	C	-0.2208	0.2742	Ref 3
rs6818397	4:3434885	rs6818397	RGS12	G	T	-0.03119	0.489	Ref 3
rs6831256	4:3473139	rs6831256	DOK7	G	A	0.01883	0.3628	Ref 2
rs6882076	5:156390297	rs6882076	TIMD4	C	T	0.04951	0.2672	Ref 3
rs6905288	6:43758873	rs6905288	VEGFA	A	G	0.03259	0.2751	Ref 2
rs72654473	19:45414399	rs445925	APOC1	A	C	0.1431	0.08517	Ref 3
rs738409	22:44324727	rs738409	PNPLA3	G	C	-0.0386807	0.4536	Ref 3
rs7499892	16:57006590	rs7499892	CETP	T	C	0.02476	0.161	Ref 3
rs769449	19:45410002	rs769449	APOE	A	G	0.1031012	0.08447	Ref 3
rs7897379	10:65301725	rs7897379	REEP3	C	T	-0.02425	0.3738	Ref 2
rs995000	1:63107526	rs995000	DOCK7	T	C	-0.07119	0.1948	Ref 2
LDL-C								
rs11125936	2:62871225	rs11125936	AC092155.4	C	T	-0.05171	0.2108	Ref 3
rs11136341	8:145043543	rs11136341	PLEC	A	G	-0.0334	0.1087	Ref 2
rs1129555	10:113910721	rs1129555	GPAM	G	A	-0.02618	0.3006	Ref 2
rs11557092	19:11257018	rs11557092	SPC24	C	T	0.0674	0.2574	Ref 3
rs1169288	12:121416650	rs1169288	HNF1A	C	A	0.0335517	0.4062	Ref 3
rs117711462	1:93159927	rs117711462	EVI5	A	G	0.210124	0.006603	Ref 3
rs12027135	1:25775733	rs12027135	TMEM57	T	A	0.01835	0.2939	Ref 2
rs12453914	17:67138878	rs12453914	ABCA6	A	C	0.01966	0.4431	Ref 1
rs12740374	1:109817590	rs12740374	CELSR2	T	G	-0.1772846	0.0599	Ref 3
rs12927205	16:72025077	rs12927205	PKD1L3	A	G	0.0711	0.2632	Ref 1
rs13277801	8:59353534	rs13277801	UBXN2B	T	C	-0.0348	0.2025	Ref 2
rs13306194	2:21252534	rs13306194	APOB	A	G	-0.09929	0.1193	Ref 3
rs1367117	2:21263900	rs1367117	APOB	A	G	0.06198	0.1271	Ref 3
rs151193009	1:55509585	rs151193009	PCSK9	T	C	-0.542453	0.01422	Ref 3
rs17135399	11:126218541	rs17135399	DCPS	G	A	0.02924	0.09335	Ref 2
rs17358402	16:71967927	rs17358402	PKD1L3	T	C	0.0845382	0.05423	Ref 3
rs191835914	5:74646765	rs191835914	HMGCR	C	A	-0.1903	0.01866	Ref 3
rs2000999	16:72108093	rs2000999	HPR	A	G	0.04074	0.2594	Ref 3
rs200990725	19:11217315	rs200990725	LDLR	T	C	0.8824063	0.001208	Ref 3

© 2023 Li J et al. *JAMA Network Open.*

Genotyped variants	Chr: Pos_hg19	Initially selected variants	Gene	Effect allele	Other allele	Effect size[a]	MAF in cohort	Reference for variant selection
rs2081687	8:59388565	rs2081687	CYP7A1	C	T	-0.03555	0.211	Ref 3
rs2328223	20:17845921	rs2328223	RIN2	A	C	-0.0379	0.2051	Ref 2
rs2642442	1:220973563	rs2642442		T	C	0.0444	0.1699	Ref 2
rs312949	2:21334283	rs312949	TDRD15	C	G	0.0381	0.277	Ref 1
rs3846663	5:74655726	rs3846663	HMGCR	T	C	0.05841	0.4702	Ref 3
rs4302748	7:36191699	rs4302748	EEPD1	A	G	0.02305	0.0868	Ref 3
rs507666	9:136149399	rs507666	ABO	A	G	0.05168	0.2075	Ref 3
rs579459	9:136154168	rs579459	ABO	C	T	0.0512	0.2072	Ref 2
rs58542926	19:19379549	rs58542926	TM6SF2	T	C	-0.03839	0.07182	Ref 3
rs6065311	20:39724338	rs6065311	TOP1	C	T	0.03584	0.1771	Ref 2
rs6871667	5:74604742	rs6871667	JMY	A	G	0.05531	0.4422	Ref 1
rs6882076	5:156390297	rs6882076	TIMD4	C	T	0.05477	0.2672	Ref 3
rs7185272	16:72013797	rs7185272	PKD1L3	C	G	0.0692	0.256	Ref 3
rs7258950	19:11250139	rs7258950	SPC24	G	A	0.06699	0.2181	Ref 1
rs7306523	12:53393964	rs7306523	EIF4B	G	A	-0.02384	0.3035	Ref 3
rs737337	19:11347493	rs737337	DOCK6	C	T	-0.03363	0.2733	Ref 3
rs7499892	16:57006590	rs7499892	CETP	T	C	-0.0369	0.161	Ref 3
rs7525649	1:55499156	rs7525649	PCSK9	T	C	0.0614	0.349	Ref 1
rs769449	19:45410002	rs769449	APOE	A	G	0.1462	0.08447	Ref 3
rs7770628	6:161018174	rs7770628	LPA	T	C	-0.067	0.1133	Ref 3
rs7901016	10:74637326	rs7901016	MCU	C	T	-0.0439231	0.2765	Ref 3
rs7965082	12:100800193	rs7965082	SLC17A8	T	C	-0.035	0.4124	Ref 3
rs9357121	6:31240479	rs9357121	HLA-B	T	G	0.0391	0.171	Ref 1
rs9390698	6:101296389	rs9390698	ASCC3	A	G	0.01757	0.2708	Ref 3
rs9534262	13:32936646	rs9534262	BRCA2	T	C	0.03	0.4601	Ref 2
HDL-C								
rs10096633	8:19830921	rs10096633	LPL	T	C	0.1474	0.09916	Ref 3
rs10773003	12:123775127	rs10773003	SBNO1	A	G	0.03458	0.2806	Ref 2
rs1077834	15:58723479	rs1077834	LIPC	C	T	0.1322	0.3966	Ref 2
rs11869286	17:37813856	rs11869286	STARD3	C	G	0.0245728	0.4321	Ref 2
rs12718465	11:116707736	rs12718465	APOA1	T	C	-0.0863	0.03293	Ref 3
rs12801636	11:65391317	rs12801636	PCNX3	A	G	0.0333	0.4334	Ref 2
rs12970066	18:47107152	rs12970066	LIPG	G	C	0.04582	0.3003	Ref 1
rs13702	8:19824492	rs13702	LPL	C	T	0.1219	0.1992	Ref 3
rs148910227	7:80302116	rs148910227	CD36	T	C	0.342	0.003625	Ref 3
rs1532085	15:58683366	rs1532085		G	A	-0.1125	0.4596	Ref 3
rs1689800	1:182168885	rs1689800	LINC01344	G	A	-0.02752	0.2945	Ref 2
rs17145738	7:72982874	rs17145738	TBL2	T	C	0.04545	0.1093	Ref 2
rs174546	11:61569830	rs174546	FADS1	T	C	-0.0457092	0.4576	Ref 2
rs174547	11:61570783	rs174547	FADS1	C	T	-0.0458525	0.4578	Ref 3
rs17695224	19:52324216	rs17695224	FPR3	A	G	-0.03669	0.2003	Ref 2

© 2023 Li J et al. *JAMA Network Open.*

Genotyped variants	Chr: Pos_hg19	Initially selected variants	Gene	Effect allele	Other allele	Effect size[a]	MAF in cohort	Reference for variant selection
rs1800588	15:58723675	rs1800588	LIPC	T	C	0.1312	0.3788	Ref 3
rs180327	11:116623659	rs180327	BUD13	T	C	0.1022	0.3411	Ref 3
rs181359	22:21928641	rs181359	UBE2L3	A	G	-0.03879	0.4829	Ref 3
rs181360	22:21928916	rs181360	UBE2L3	G	T	-0.03879	0.4829	Ref 2
rs1883025	9:107664301	rs1883025	ABCA1	T	C	-0.1081	0.222	Ref 3
rs2000813	18:47093864	rs2000813	LIPG	T	C	0.04374	0.3031	Ref 3
rs2043085	15:58680954	rs2043085	ALDH1A2	C	T	-0.1081	0.4645	Ref 3
rs2066714	9:107586753	rs2066714	ABCA1	C	T	0.04352	0.2808	Ref 3
rs2068888	10:94839642	rs2068888	CYP26A1	A	G	0.03094	0.1821	Ref 3
rs2075291	11:116661392	rs2075291	APOA5	A	C	-0.2577	0.05777	Ref 3
rs2156552	18:47181668	rs2156552	SMUG1P1	T	A	0.06471	0.1814	Ref 2
rs2230808	9:107562804	rs2230808	ABCA1	C	T	0.04999	0.38	Ref 3
rs2245019	8:116622906	rs2245019	TRPS1	A	C	0.04498	0.1411	Ref 2
rs2292318	16:67985706	rs2292318	SLC12A4	T	C	0.06973	0.1151	Ref 2
rs2296172	1:39835817	rs2296172	MACF1	A	G	0.0334	0.1739	Ref 2
rs2297991	10:113913222	rs2297991	GPAM	C	T	-0.04947	0.301	Ref 3
rs2303790	16:57017292	rs2303790	CETP	G	A	0.4241	0.02346	Ref 3
rs247616	16:56989590	rs247616	AC012181.1	T	C	0.2441	0.163	Ref 3
rs2575876	9:107665739	rs2575876	ABCA1	A	G	-0.1078	0.2192	Ref 1
rs2925979	16:81534790	rs2925979	CMIP	C	T	0.03966	0.4121	Ref 3
rs2972143	2:227116365	rs2972143	NEU2	A	G	0.0593	0.07838	Ref 2
rs326214	11:47298360	rs326214	MADD	A	G	-0.0372	0.3242	Ref 2
rs3785100	16:67997920	rs3785100	SLC12A4	C	T	0.06773	0.1154	Ref 3
rs4129767	17:76403984	rs4129767	PGS1	A	G	0.0201	0.3404	Ref 2
rs4142995	7:17919258	rs4142995	SNX13	T	G	-0.0409	0.4439	Ref 2
rs4148008	17:66875294	rs4148008	ABCA8	C	G	0.0252	0.4312	Ref 2
rs439401	19:45414451	rs439401	APOC1	T	C	0.0083	0.4191	Ref 3
rs4883263	12:7649484	rs4883263	CD163	C	T	-0.0466099	0.3107	Ref 3
rs4917014	7:50305863	rs4917014	AC020743.3	G	T	0.02675	0.3069	Ref 2
rs4939883	18:47167214	rs4939883	SMUG1P1	C	T	0.0639343	0.1889	Ref 3
rs499974	11:75455021	rs499974	RN7SL786P	A	C	-0.03008	0.2397	Ref 2
rs634501	5:180218668	rs634501	MGAT1	G	A	-0.0230618	0.4777	Ref 3
rs651821	11:116662579	rs651821	APOA5	T	C	0.1428	0.2742	Ref 3
rs671	12:112241766	rs671	ALDH2	A	G	-0.047574	0.1975	Ref 3
rs6905288	6:43758873	rs6905288	VEGFA	A	G	-0.03305	0.2751	Ref 2
rs702485	7:6449272	rs702485		A	G	-0.0512	0.131	Ref 2
rs7134594	12:110000193	rs7134594	MMAB	T	C	0.03552	0.3013	Ref 2
rs7208487	17:37543449	rs7208487	FBXL20	G	T	-0.0398418	0.2632	Ref 3
rs737337	19:11347493	rs737337	DOCK6	C	T	-0.06691	0.2733	Ref 3
rs7499892	16:57006590	rs7499892	CETP	T	C	-0.1624	0.161	Ref 3
rs769449	19:45410002	rs769449	APOE	A	G	-0.1075	0.08447	Ref 3

© 2023 Li J et al. *JAMA Network Open.*

Genotyped variants	Chr: Pos_hg19	Initially selected variants	Gene	Effect allele	Other allele	Effect size[a]	MAF in cohort	Reference for variant selection
rs838880	12:125261593	rs838880	SCARB1	T	C	-0.047	0.4947	Ref 2
rs884366	6:109574095	rs884366	CCDC162P	A	G	-0.02728	0.373	Ref 3
rs9593	12:109994870	rs9593	MMAB	T	A	0.03728	0.3015	Ref 3

Abbreviations: TC, total cholesterol; TG, triglyceride; LDL-C, low-density lipoprotein cholesterol; HDL-C, high-density lipoprotein cholesterol; MAF, minor allele frequency.

a, effect sizes of variants were determined according to previous studies (Nat Genet 2017, 49(12): 1722-30; Hum Mol Genet 2017, 26(9): 1770-84; Nat Genet 2018; 50(3): 390-400.).

Ref 1, Circ Cardiovasc Genet 2016, 9(1): 37-44; Ref 2, Hum Mol Genet 2017, 26(9): 1770-1784; Ref 3, Nat Genet 2017, 49(12): 1722-1730.

© 2023 Li J et al. *JAMA Network Open.*

eTable 3. Sample Sizes According to Sex, Age and Polygenic Risk Group

	Polygenic risk in males				Polygenic risk in females			
	Low	Intermediate	High	Total	Low	Intermediate	High	Total
TC								
<40	524	1472	495	2491	714	2125	711	3550
40-49	937	2818	904	4659	1328	3973	1309	6610
50-59	968	2945	998	4911	1352	3998	1308	6658
≥60	703	2164	736	3603	936	2897	1002	4835
All ages	3132	9399	3133	15 664	4330	12 993	4330	2 1653
TG								
<40	531	1494	466	2491	740	2105	705	3550
40-49	945	2827	887	4659	1353	3942	1315	6610
50-59	954	2942	1015	4911	1294	4005	1359	6658
≥60	702	2136	765	3603	943	2941	951	4835
All ages	3132	9399	3133	15 664	4330	12 993	4330	21 653
LDL-C								
<40	492	1530	469	2491	724	2135	691	3550
40-49	922	2820	917	4659	1295	3972	1343	6610
50-59	974	2938	999	4911	1371	3982	1305	6658
≥60	744	2111	748	3603	940	2904	991	4835
All ages	3132	9399	3133	15 664	4330	12 993	4330	21 653
HDL-C								
<40	495	1497	499	2491	678	2136	736	3550
40-49	917	2799	943	4659	1347	3951	1312	6610
50-59	970	2958	983	4911	1342	3997	1319	6658
≥60	750	2145	708	3603	963	2909	963	4835
All ages	3132	9399	3133	15 664	4330	12 993	4330	21 653

Abbreviations: TC, total cholesterol; TG, triglyceride; LDL-C, low-density lipoprotein cholesterol; HDL-C, high-density lipoprotein cholesterol.

© 2023 Li J et al. *JAMA Network Open.*

eTable 4. Multivariable-Adjusted Estimated Annual Changes of Lipid (mg/dL) and 95% CIs According to Polygenic Risk Group Among Participants Without Lipid Treatment

Blood lipid	Polygenic risk			*P* for trend
	Low	Intermediate	High	
Total				
TC	0.00 (-0.09, 0.09)	0.77 (0.71, 0.82)	1.30 (1.21, 1.39)	<.001
TG	-1.38 (-1.59, -1.17)	0.37 (0.22, 0.51)	3.66 (3.32, 4.00)	<.001
LDL-C	-0.01 (-0.08, 0.07)	0.53 (0.48, 0.57)	0.86 (0.77, 0.94)	<.001
HDL-C	-0.11 (-0.14, -0.07)	0.20 (0.18, 0.22)	0.52 (0.48, 0.56)	<.001
Males				
TC	-0.47 (-0.60, -0.34)	0.34 (0.27, 0.42)	0.89 (0.75, 1.02)	<.001
TG	-1.91 (-2.25, -1.58)	-0.31 (-0.54, -0.09)	3.04 (2.51, 3.57)	<.001
LDL-C	-0.26 (-0.37, -0.15)	0.22 (0.16, 0.29)	0.64 (0.52, 0.77)	<.001
HDL-C	-0.13 (-0.19, -0.07)	0.16 (0.12, 0.19)	0.49 (0.43, 0.55)	<.001
Females				
TC	0.37 (0.25, 0.49)	1.10 (1.03, 1.17)	1.62 (1.50, 1.75)	<.001
TG	-0.97 (-1.24, -0.70)	0.90 (0.72, 1.09)	4.22 (3.77, 4.66)	<.001
LDL-C	0.19 (0.09, 0.29)	0.76 (0.70, 0.82)	1.02 (0.91, 1.13)	<.001
HDL-C	-0.09 (-0.13, -0.04)	0.23 (0.21, 0.26)	0.54 (0.49, 0.59)	<.001

Abbreviations: TC, total cholesterol; TG, triglyceride; LDL-C, low-density lipoprotein cholesterol; HDL-C, high-density lipoprotein cholesterol.

Adjusted for sex (only for total population), region, area, subcohort, age, education level, lipid level, smoking, alcohol consumption, body mass index, physical activity, diet, and survey year at the beginning of two adjacent examinations.

The low, intermediate, and high polygenic risk were defined as the first, second to fourth, and fifth quintiles of polygenic risk scores.

SI conversion factor: To convert TC, LDL-C, and HDL-C to mmol/L, multiply by 0.0259; to convert TG to mmol/L, multiply by 0.0113.

© 2023 Li J et al. *JAMA Network Open.*

eFigure 1. Flow Chart of Study Participants Included and Excluded in the Analysis

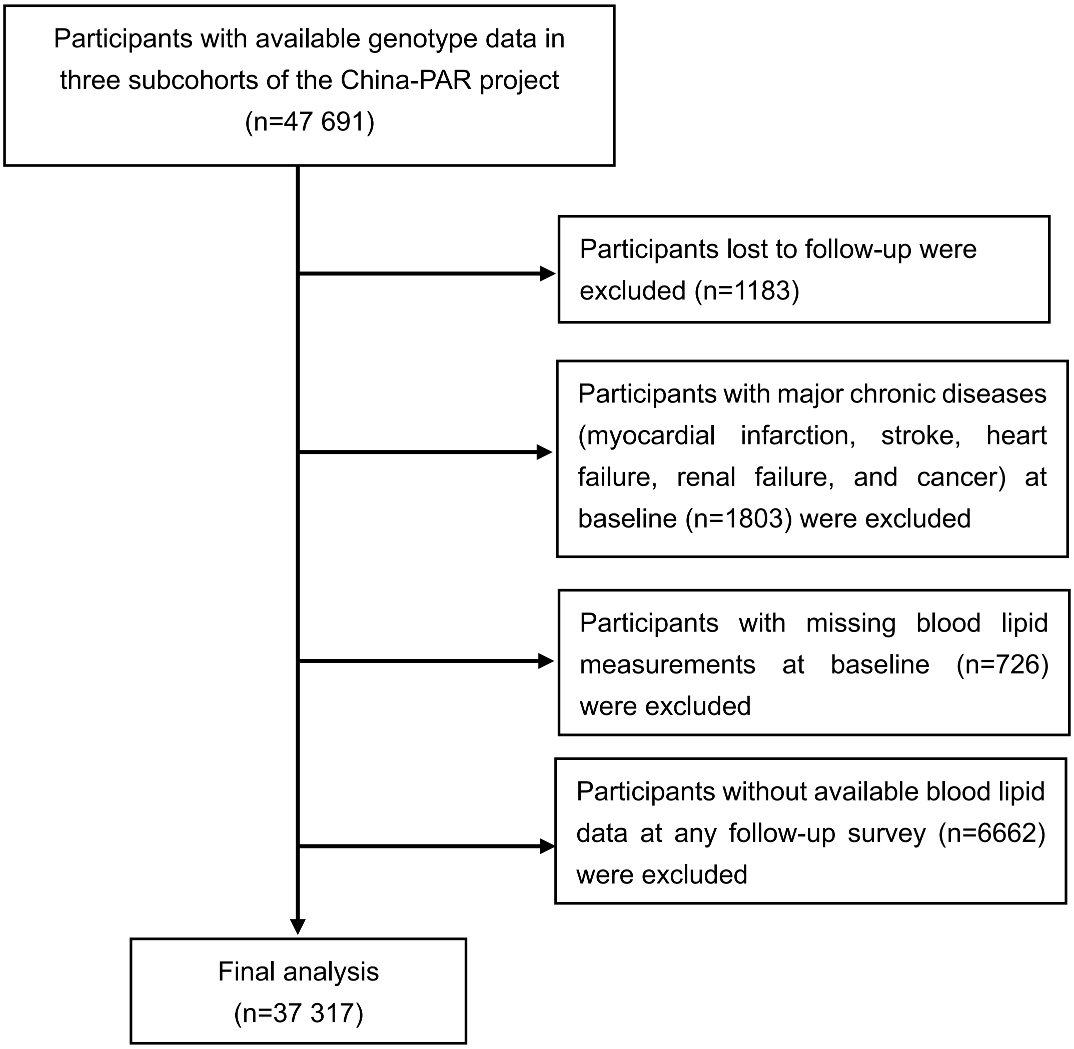

China-PAR: Prediction for Atherosclerotic Cardiovascular Disease Risk in China

© 2023 Li J et al. *JAMA Network Open.*

eFigure 2. Blood Lipid Levels at the Beginning of any 2 Adjacent Examinations

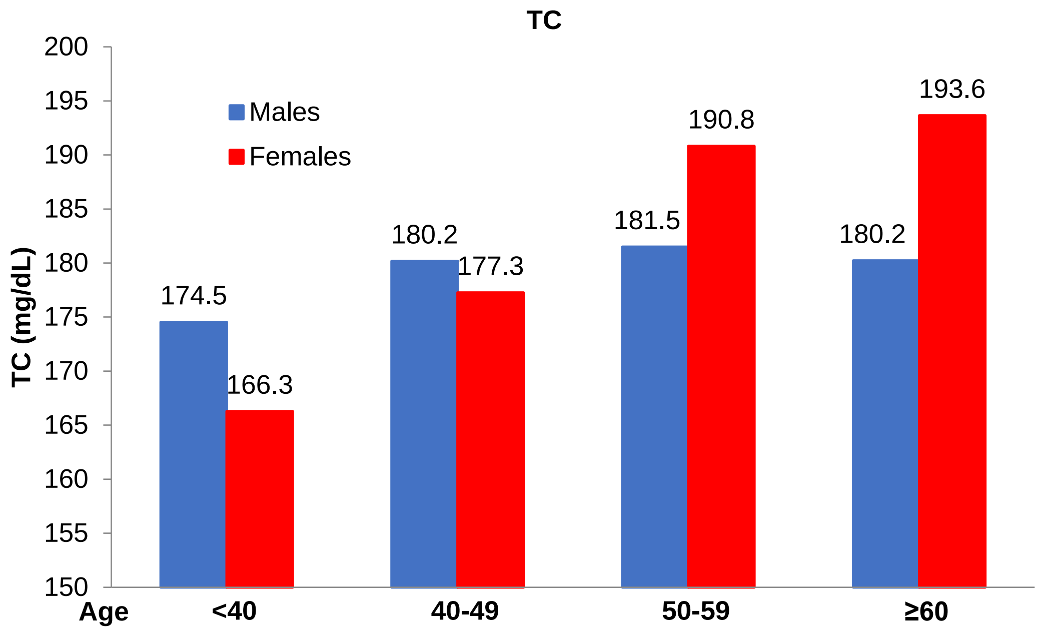

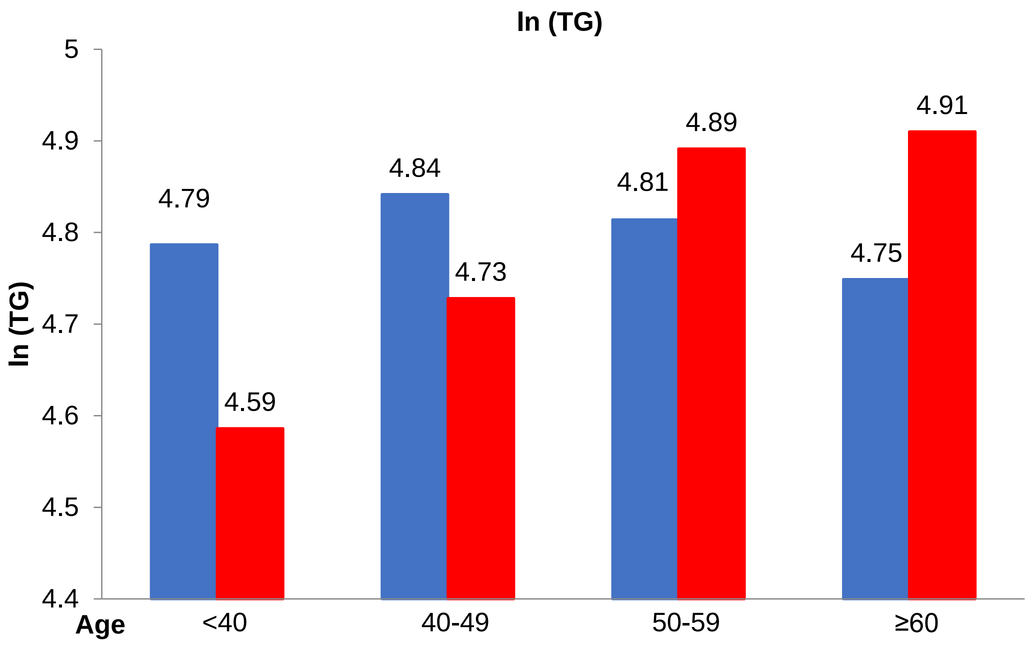

© 2023 Li J et al. *JAMA Network Open.*

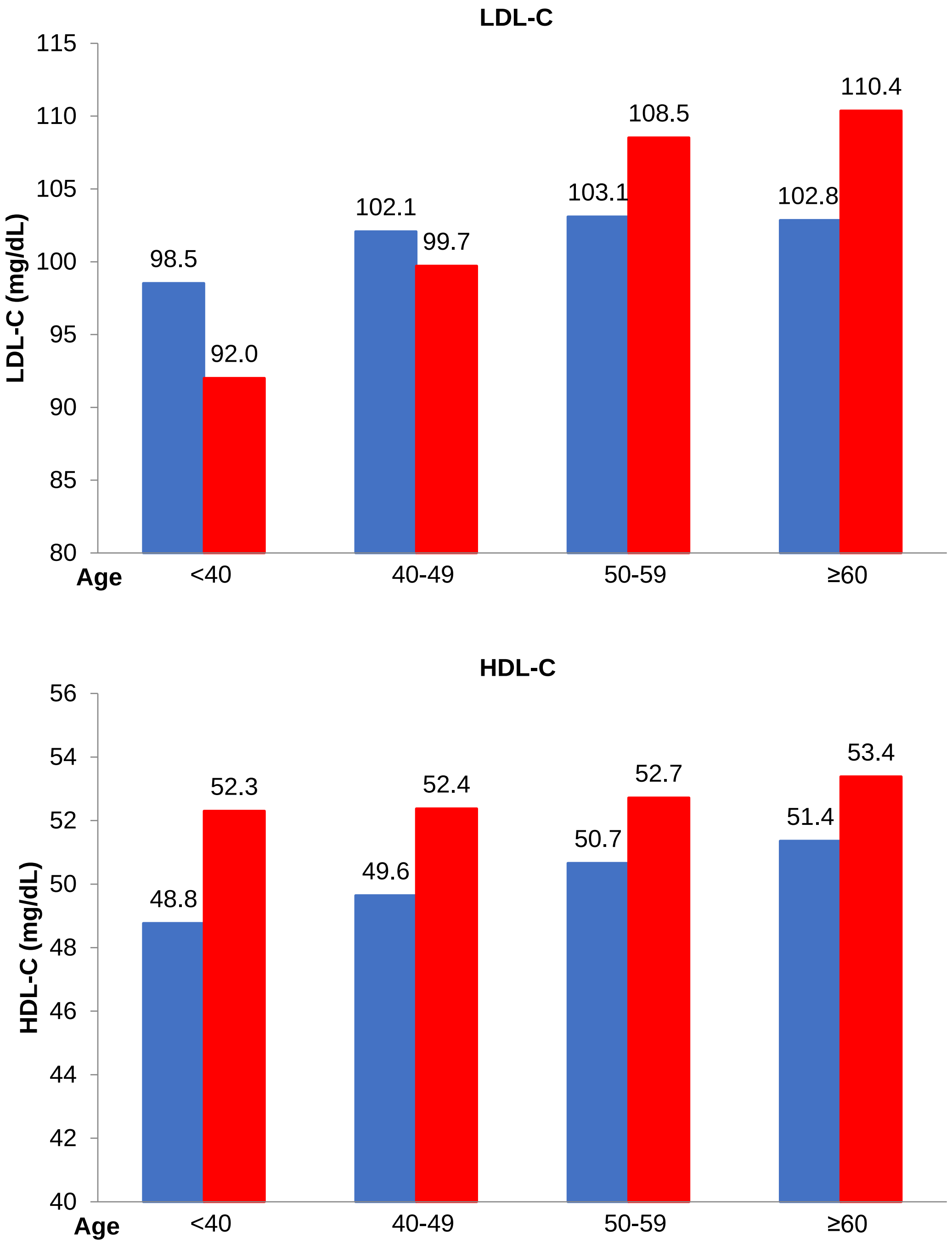

Abbreviations: TC, total cholesterol; TG, triglyceride; LDL-C, low-density lipoprotein cholesterol; HDL-C, high-density lipoprotein cholesterol; ln, natural log-transformed.

SI conversion factor: To convert TC, LDL-C, and HDL-C to mmol/L, multiply by 0.0259; to convert TG to mmol/L, multiply by 0.0113.

© 2023 Li J et al. *JAMA Network Open.*

eFigure 3. Multivariable-Adjusted Estimated Annual Changes of Lipid by Quintiles of Polygenic Risk

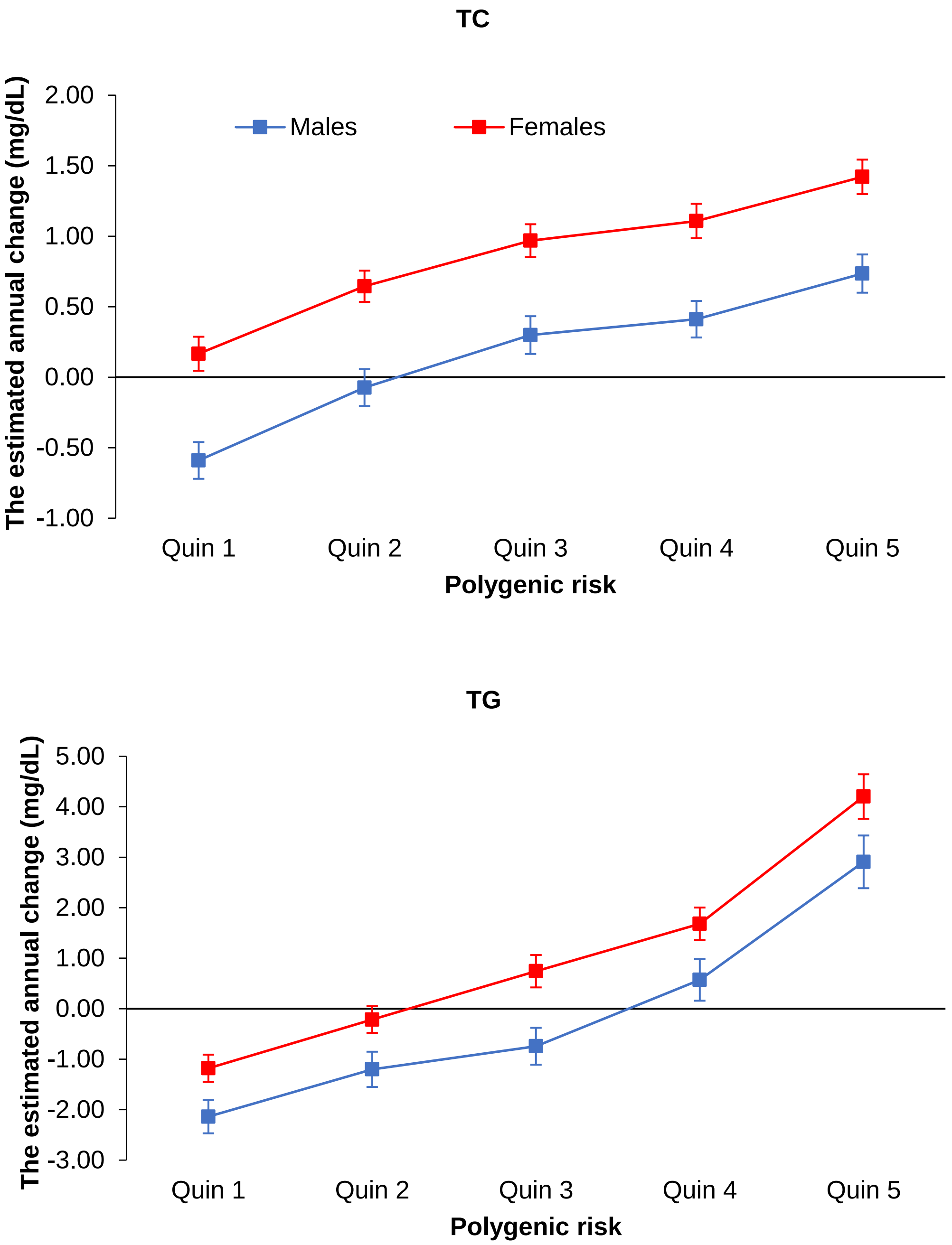

© 2023 Li J et al. *JAMA Network Open.*

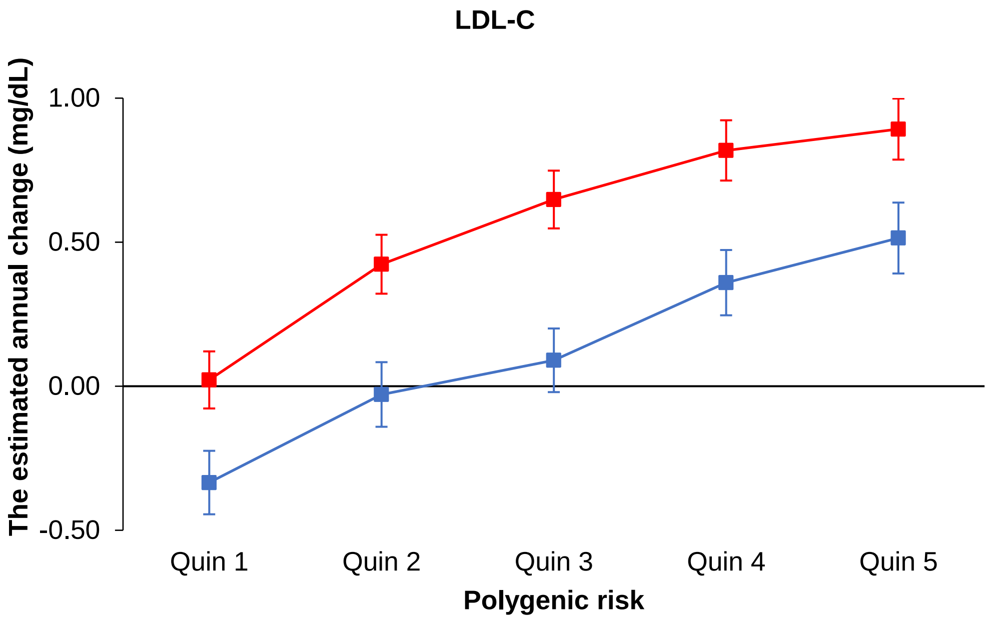

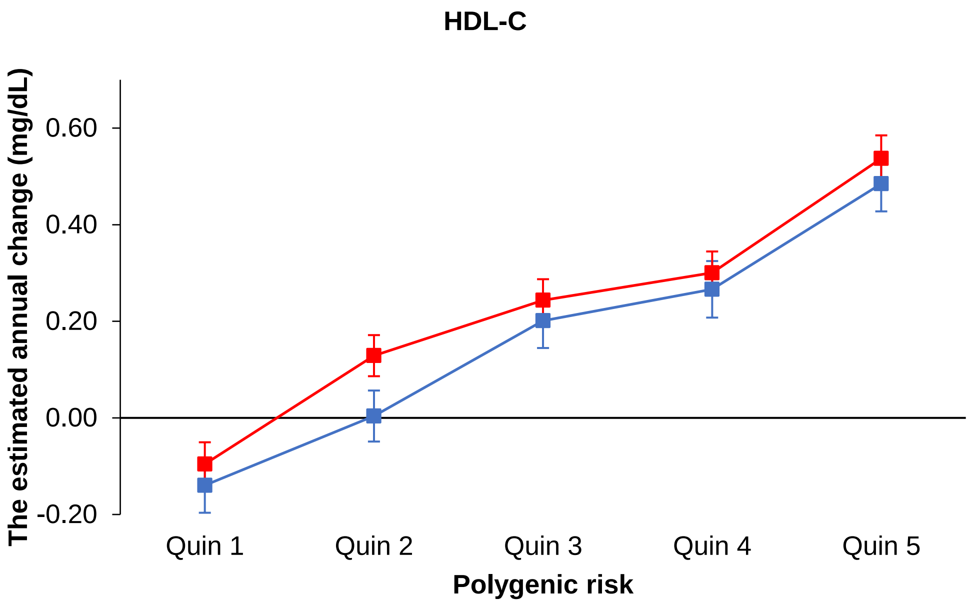

Abbreviations: TC, total cholesterol; TG, triglyceride; LDL-C, low-density lipoprotein cholesterol; HDL-C, high-density lipoprotein cholesterol; Quin, quintile.

Adjusted for region, area, subcohort, age, education level, lipid level, smoking, alcohol consumption, body mass index, physical activity, diet, and survey year at the beginning of two adjacent examinations.

Dots are the estimated annual changes of lipid, and vertical lines represent the 95% confidence intervals.

All *P* values for trend were <.001.

SI conversion factor: To convert TC, LDL-C, and HDL-C to mmol/L, multiply by 0.0259; to convert TG to mmol/L, multiply by 0.0113.

© 2023 Li J et al. *JAMA Network Open.*

eFigure 4. Multivariable-Adjusted Estimated Annual Changes of Lipid by Age Group Among Participants Without Lipid Treatment

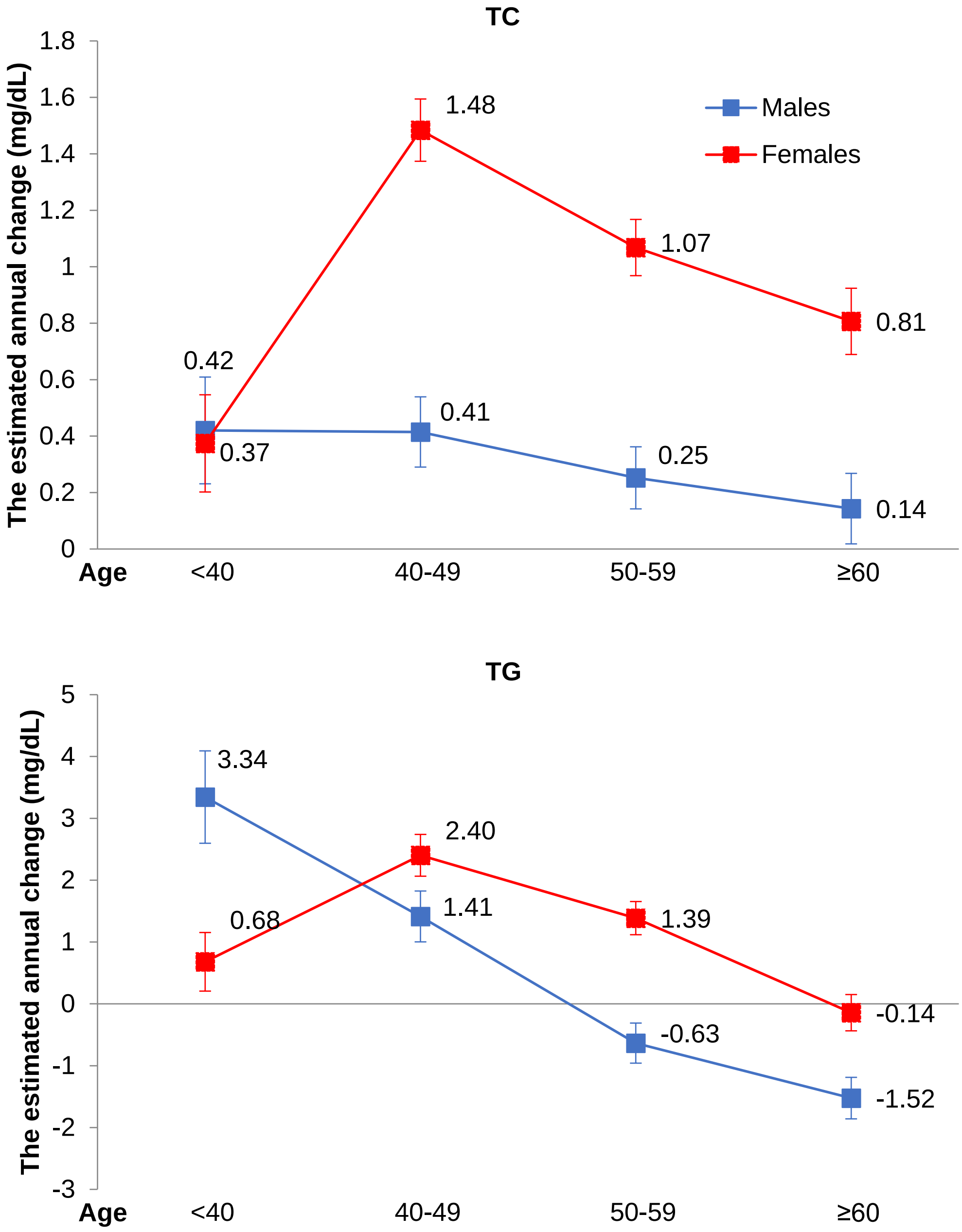

© 2023 Li J et al. *JAMA Network Open.*

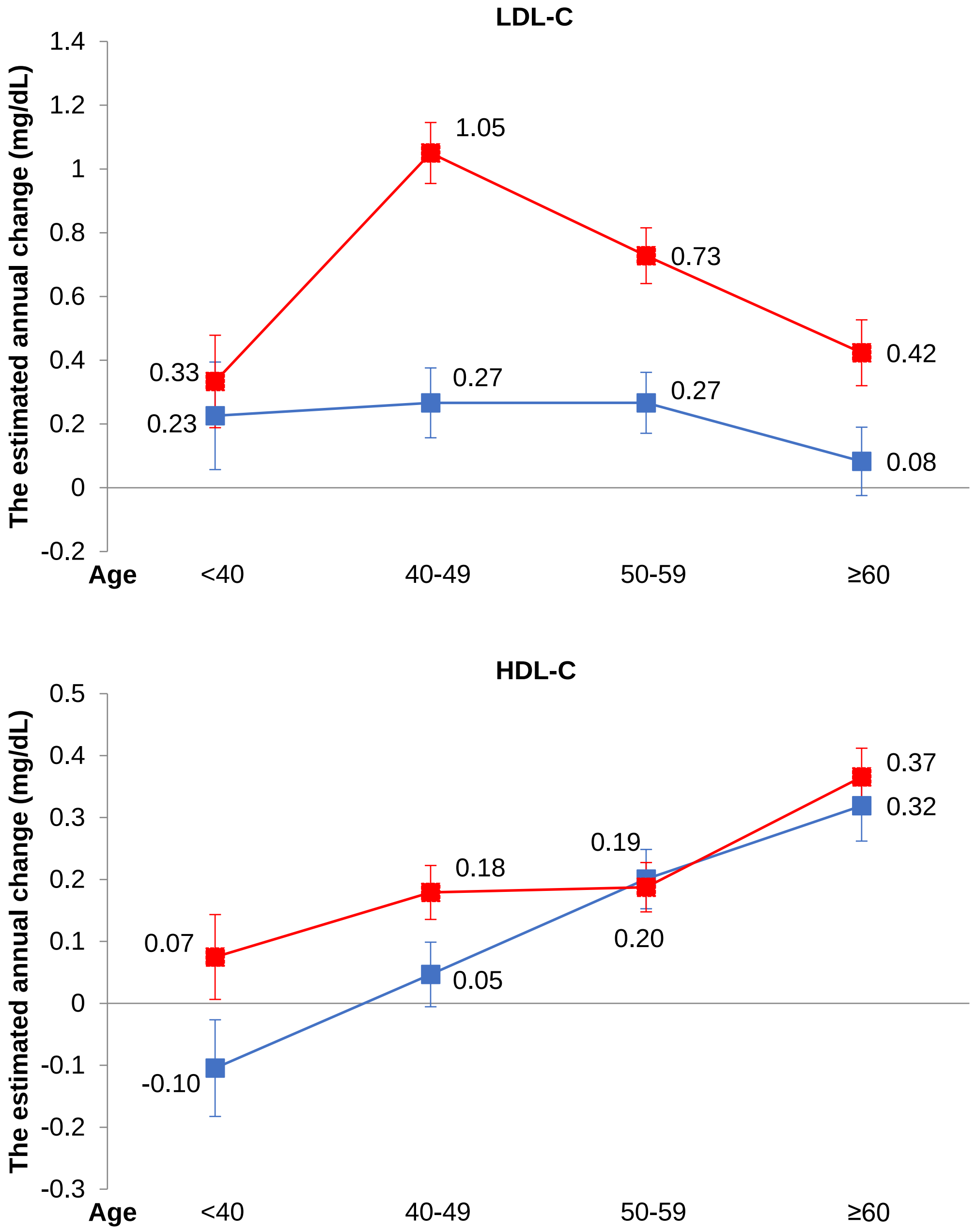

Abbreviations: TC, total cholesterol; TG, triglyceride; LDL-C, low-density lipoprotein cholesterol; HDL-C, high-density lipoprotein cholesterol.

Adjusted for region, area, subcohort, education level, lipid level, smoking, alcohol consumption, body mass index, physical activity, diet, and survey year at the beginning of two adjacent examinations.

Dots are the estimated annual changes of lipid, and vertical lines represent the 95% confidence intervals.

SI conversion factor: To convert TC, LDL-C, and HDL-C to mmol/L, multiply by 0.0259; to convert TG to mmol/L, multiply by 0.0113.

© 2023 Li J et al. *JAMA Network Open.*

eFigure 5. Multivariable-Adjusted Estimated Annual Changes of Lipid According to Polygenic Risk and Age Group Among Males Without Treatment for Lipid

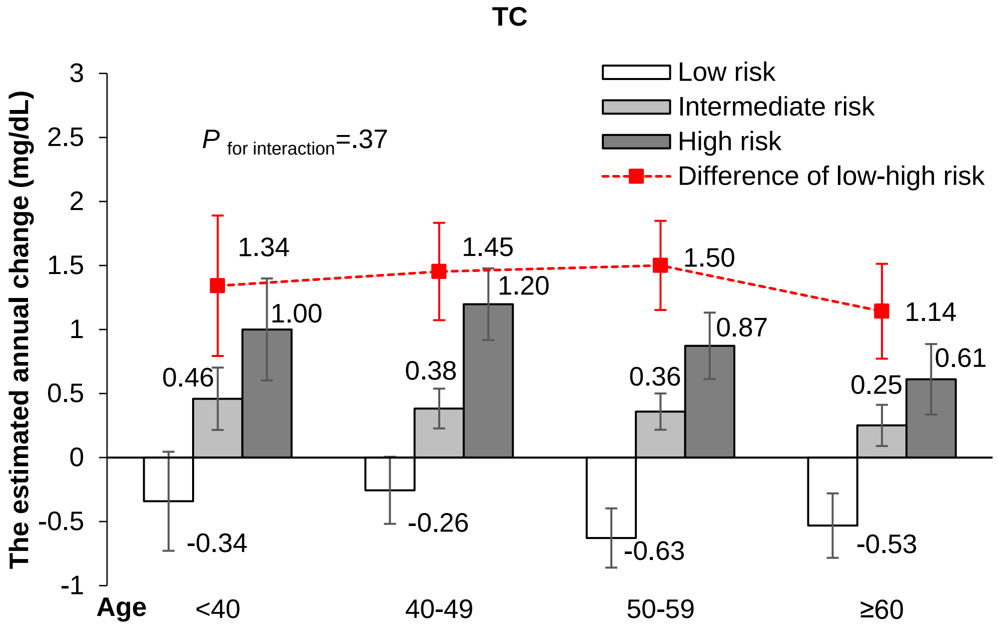

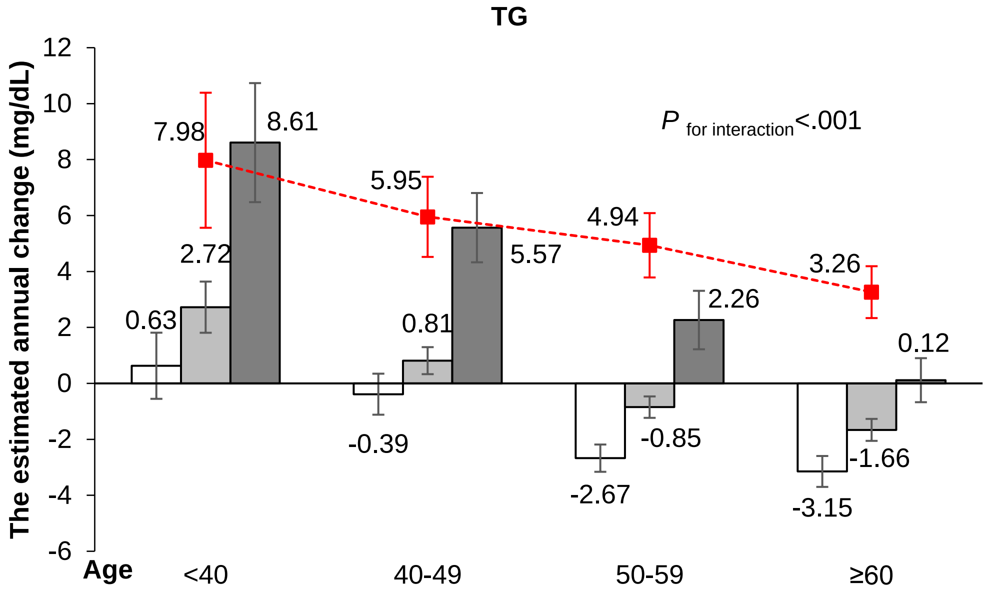

© 2023 Li J et al. *JAMA Network Open.*

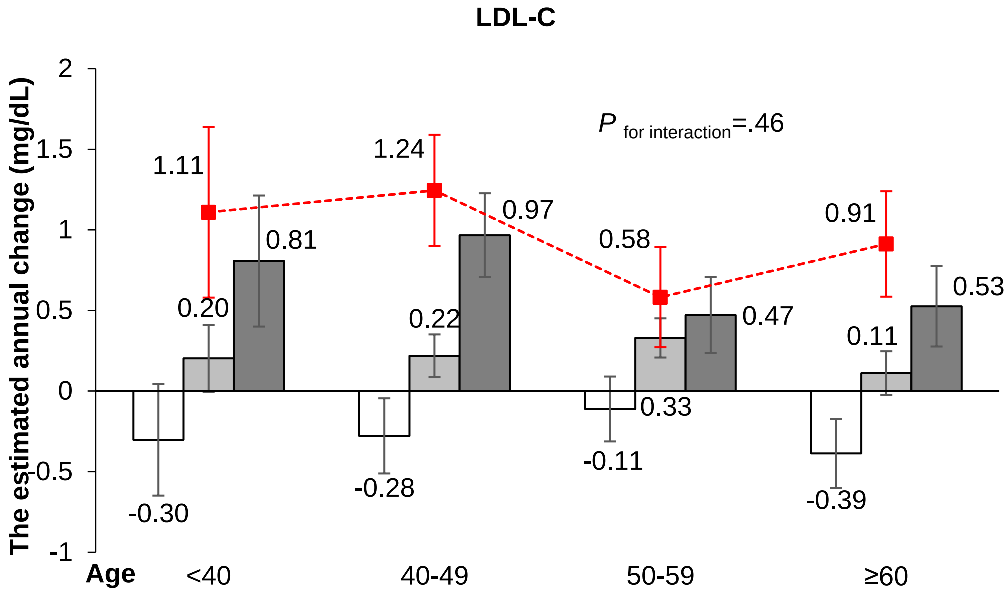

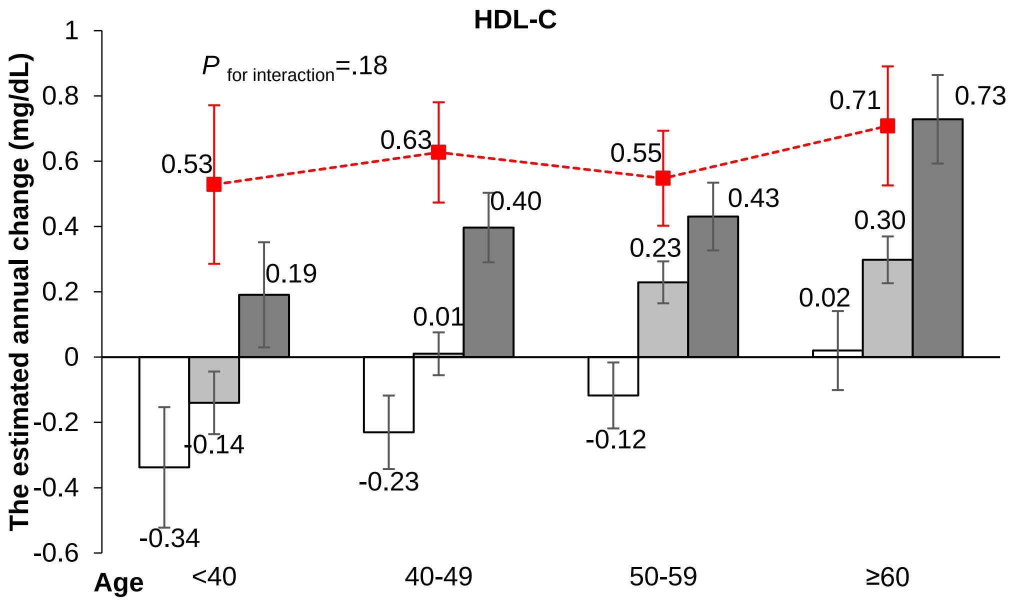

Abbreviations: TC, total cholesterol; TG, triglyceride; LDL-C, low-density lipoprotein cholesterol; HDL-C, high-density lipoprotein cholesterol.

Adjusted for region, area, subcohort, education level, lipid level, smoking, alcohol consumption, body mass index, physical activity, diet, and survey year at the beginning of two adjacent examinations.

The low, intermediate, and high polygenic risk were defined as the first, second to fourth, and fifth quintiles of polygenic risk scores. Bar are the estimated annual changes, dots are differences of the estimated annual changes between low and high polygenic risk, and vertical lines represent the 95% confidence intervals.

SI conversion factor: To convert TC, LDL-C, and HDL-C to mmol/L, multiply by 0.0259; to convert TG to mmol/L, multiply by 0.0113.

© 2023 Li J et al. *JAMA Network Open.*

eFigure 6. Multivariable-Adjusted Estimated Annual Changes of Lipid According to Polygenic Risk and Age Group Among Females Without Lipid Treatment

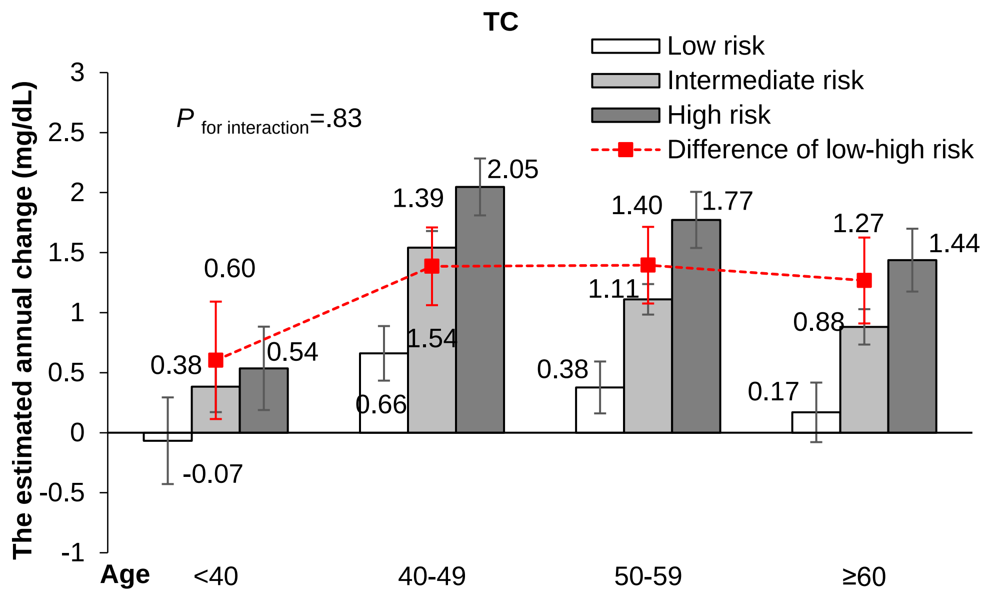

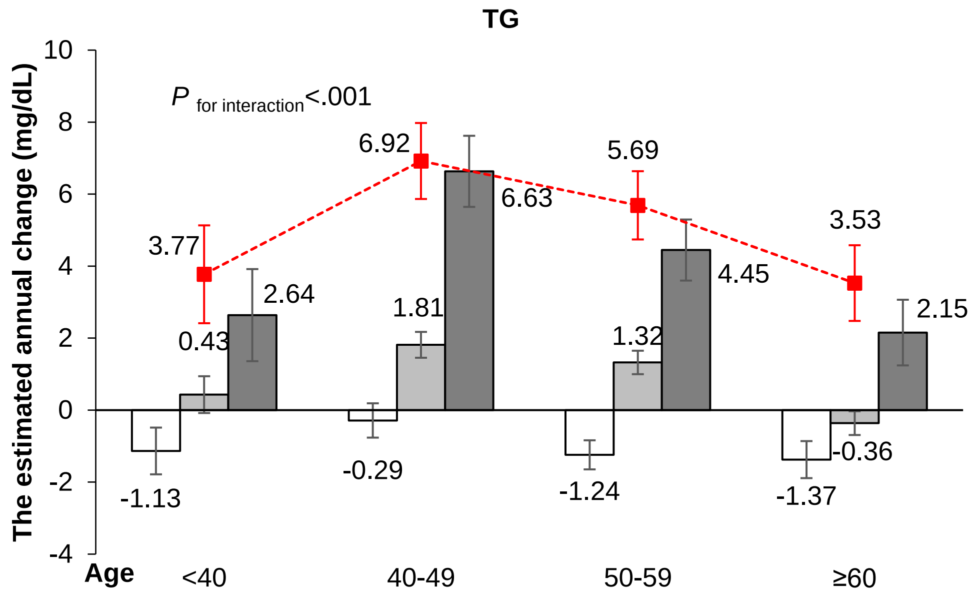

© 2023 Li J et al. *JAMA Network Open.*

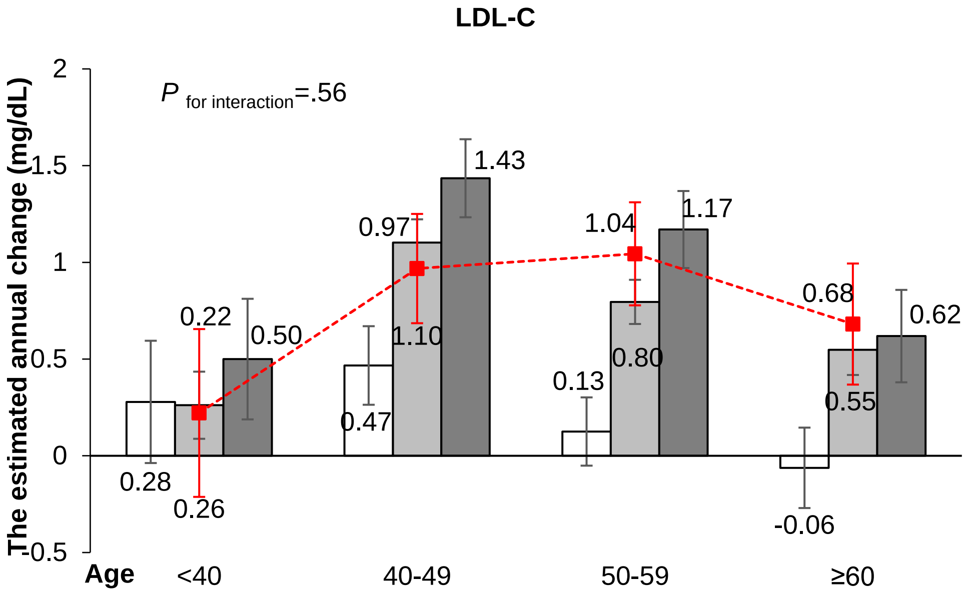

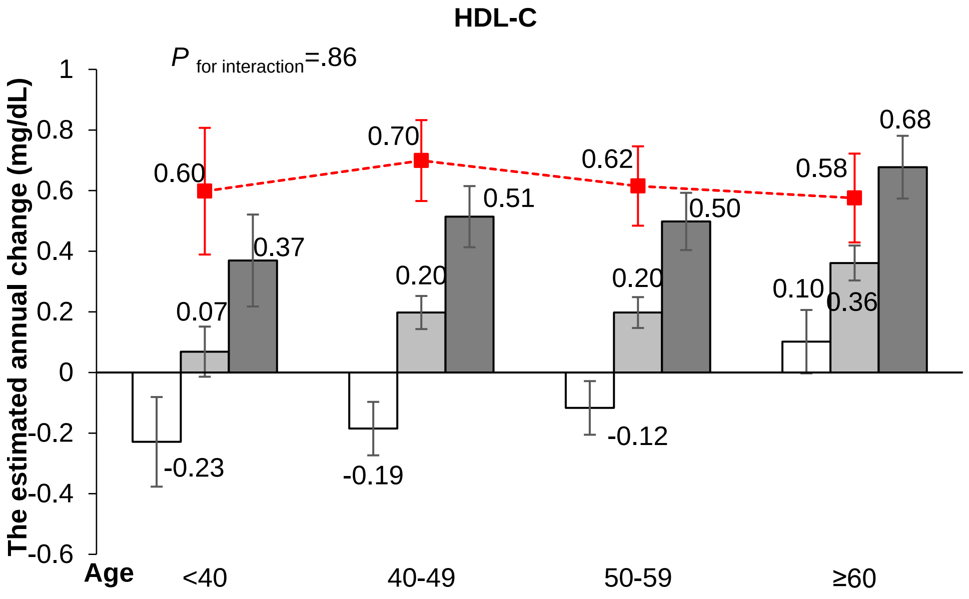

Abbreviations: TC, total cholesterol; TG, triglyceride; LDL-C, low-density lipoprotein cholesterol; HDL-C, high-density lipoprotein cholesterol.

Adjusted for region, area, subcohort, education level, lipid level, smoking, alcohol consumption, body mass index, physical activity, diet, and survey year at the beginning of two adjacent examinations.

The low, intermediate, and high polygenic risk were defined as the first, second to fourth, and fifth quintiles of polygenic risk scores. Bar are the estimated annual changes, dots are differences of the estimated annual changes between low and high polygenic risk, and vertical lines represent the 95% confidence intervals.

SI conversion factor: To convert TC, LDL-C, and HDL-C to mmol/L, multiply by 0.0259; to convert TG to mmol/L, multiply by 0.0113.

© 2023 Li J et al. *JAMA Network Open.*
