# Supplementary material for: Age and Genetic Risk Score and Rates of Blood Lipid Changes in China
Source: JAMA Netw Open. 2023 Mar 29;6(3):e235565. doi: 10.1001/jamanetworkopen.2023.5565 (PMC10061238; doi:10.1001/jamanetworkopen.2023.5565)
Supplement: Supplement 2. — Data Sharing Statement [file jamanetwopen-e235565-s002.pdf]

Data Sharing Statement

Li. Age and Genetic Risk Score and Rates of Blood Lipid Changes in China. *JAMA Netw Open*. Published March 29, 2023. doi:10.1001/jamanetworkopen.2023.5565

Data
Data available: Yes
Data types: Deidentified participant data, Data dictionary
How to access data: luxf@pumc.edu.cn
When available: With publication

Supporting Documents
Document types: None

Additional Information
Who can access the data: Xiangfeng Lu
Types of analyses: For results verification
Mechanisms of data availability: with a signed data access agreement
